# Early detection and diagnosis of cancer with interpretable machine learning to uncover cancer-specific DNA methylation patterns

**DOI:** 10.1093/biomethods/bpae028

**Published:** 2024-06-20

**Authors:** Izzy Newsham, Marcin Sendera, Sri Ganesh Jammula, Shamith A Samarajiwa

**Affiliations:** MRC Cancer Unit, University of Cambridge, Cambridge, CB2 0XZ, United Kingdom; MRC Biostatistics Unit, University of Cambridge, Cambridge, CB2 0SR, United Kingdom; MRC Cancer Unit, University of Cambridge, Cambridge, CB2 0XZ, United Kingdom; Jagiellonian University, Faculty of Mathematics and Computer Science, 30-348 Kraków, Poland; CRUK Cambridge Institute, University of Cambridge, Cambridge, CB2 0RE, United Kingdom; MedGenome labs, Bengaluru, 560099, India; MRC Cancer Unit, University of Cambridge, Cambridge, CB2 0XZ, United Kingdom; Imperial College London, Hammersmith Campus, London, W12 0NN, United Kingdom

## Abstract

Cancer, a collection of more than two hundred different diseases, remains a leading cause of morbidity and mortality worldwide. Usually detected at the advanced stages of disease, metastatic cancer accounts for 90% of cancer-associated deaths. Therefore, the early detection of cancer, combined with current therapies, would have a significant impact on survival and treatment of various cancer types. Epigenetic changes such as DNA methylation are some of the early events underlying carcinogenesis. Here, we report on an interpretable machine learning model that can classify 13 cancer types as well as non-cancer tissue samples using only DNA methylome data, with 98.2% accuracy. We utilize the features identified by this model to develop EMethylNET, a robust model consisting of an XGBoost model that provides information to a deep neural network that can generalize to independent data sets. We also demonstrate that the methylation-associated genomic loci detected by the classifier are associated with genes, pathways and networks involved in cancer, providing insights into the epigenomic regulation of carcinogenesis.

## Introduction

Cancer remains one of the most challenging human diseases, with over 19 million cases and 10 million deaths reported annually [1]. The increase of an ageing population worldwide, together with exposure to environmental carcinogens, and lifestyle choices such as poor diets, smoking and lack of physical activity contribute to the worldwide increase in cancer incidences. The evolutionary nature of cancer, complex interactions with the tissue micro-environment and host immune system, engender heterogeneity and make the pursuit and development of interventions difficult. Therefore, early detection and diagnosis of cancer, leading to better interventions and increased survival, remain one of the more effective avenues in combating cancer.

Each of our somatic cells contains a single identical genome, incorporating the information necessary to specify and maintain our characteristics. In contrast, each cell will exhibit multiple epigenomes that change during different cellular states and over the passage of time. These epigenomes consist of a collection of reversible chromatin structures, interactions and modifications that do not change the DNA sequence and may be heritable across progeny cells. Histone variations, post translational modifications of the amino terminal tails of histone proteins, and covalent modification of DNA are some of the factors that contribute to epigenomic change. Notably, covalent methylation of DNA is one such reversible chemical modification with many functional consequences, and evidence for its role in embryonic development, cell differentiation, genomic imprinting, X chromosome inactivation, repression of regulatory elements, genome maintenance and the regulation of gene expression has accumulated in the last few decades [2].

Aberrant DNA methylation is observed in many cancers. CpG island promoter hypermethylation of tumour suppressor genes is an early neoplastic event in many tumours [3–6]. In addition, global DNA hypomethylation can lead to chromosomal instability, activation of oncogenes and latent retrotransposons that promote carcinogenesis [7]. Hypomethylation is seen in many cancer types, including cervical, prostate, hepatocellular, breast, brain, and leukaemia [8–11]. These hyper- and hypo-methylation patterns can serve as cancer-associated signals and prognostic biomarkers. They are of particular use for early detection of cancer, as epigenetic modifications are some of the earliest neoplastic events associated with carcinogenesis [12, 13]. Computational methods that detect these complex neoplastic methylation patterns can thus assist in cancer early detection, diagnosis, and screening. Here, we developed both binary and multiclass machine learning models to identify multiple cancer types from non-cancerous tissue samples. An expanding corpus of literature supports the use of classification methods trained on DNA methylation changes to identify carcinogenic signatures. Some of the more relevant works are reviewed in Table 1. Moreover, we previously demonstrated that machine learning models, leveraging DNA methylation data from 1228 tissue samples can accurately classify pathological subtypes of renal tumours [14]. In this study, we introduce a multiclass deep neural network, EMethylNET: *Explainable Methylome Neural network for Evaluation of Tumours*. EMethylNET is robust, generalizable, and interpretable and demonstrates high predictive accuracy.

**Table 1.** Summary of related studies, including EMethylNET, detailing the model type, number of train/test data sources (and total sample number), number of external validation data sources (and total sample number), number of CpGs input into the model and number of CpGs used by the model.

Work	Model type	Train/test data sources (n)	**External validation data sources** (n)	CpGs input to the model	CpGs used by the model
Hao 2017 [18]	LASSO	1 (2676)	1 (718)		
Tang 2017 [19]	Random forest	1 (5379)	7 (504)	9-998	
Capper 2018 [20]	Random forest	1 (3905)	5 (401)	10000	
Peng 2018 [21]	LASSO	1 (1478)	3 (267)		128
Ding 2019 [22]	Logistic regression	1 (7605)	6 (742)	12	12
Zheng 2020 [23]	DNN	1 (7339)	12 (972)	10360	10360
Koelsche 2021 [24]	Random forest	1 (1077)	4 (428)	10000	
Liu 2021 [25]	XGBoost	1 (7224)	0	*≤* 294	
Modhurkur 2021 [26]	Random forest	9 (9303)	0	2978	
Ibrahim 2022 [27]	PLSDA	1 (6502)	10 (1595)	20	20
Kuschel 2022 [28]	Random forest	3 (369)	0	50000	
Zhang 2023 [29]	Linear support vector classifier	1 (781)	1 (4702)	1588	
EMethylNET	XGBoost and DNN	1 (6224)	9 (940)	276016	3388

## Materials and methods

### Microarray-based methylation analysis

Methylome microarray data were obtained from The Cancer Genome Atlas (TCGA) GDC data portal (https://www.cancer.gov/ccg/research/genome-sequencing/tcga, RRID: SCR 003193). The data sets utilized were from the Illumina Infinium Human DNA Methylation 450 platform, and 13 human cancer types with at least fifteen normal samples were analysed. Metadata was also obtained from the TCGA data portal. Supplementary Table S12 shows the number of cancer and normal samples for each cancer type.

In addition to the TCGA data, a number of data sets from independent studies were also used in model evaluation. Eight of the independent data sets were from the Illumina Infinium Human DNA Methylation 450 platform, and one (ESCA 2) was from the Illumina Infinium Methylation EPIC array platform. The number of cancer and normal samples for each independent data set is shown in Supplementary Table S13. The sources of each data set are: breast cancer (BRCA): GSE52865, colon adenocarcinoma (COAD): GSE77955 (only samples from sites colon, left colon, right colon, and sigmoid are taken), esophageal carcinoma (ESCA): GSE72874, ESCA 2: EGAD00010001822 and EGAD00010001834, head and neck squamous cell carcinoma (HNSC): GSE38266 (note that half of these samples are HPV+), kidney renal clear cell carcinoma (KIRC): GSE61441, liver hepatocellular carcinoma (LIHC): GSE75041, prostate adenocarcinoma (PRAD): project PRAD-CA from ICGC, thyroid carcinoma (THCA): GSE97466. For the COAD and THCA independent data sets, details regarding the adenoma samples were obtained from their metadata.

### Data pre-processing

TCGA data were downloaded using TCGAbiolinks package (https://bioconductor.org/packages/TCGAbiolinks/, RRID: SCR 017683) [15] in R (version 3.6.2) [16], with the following pre-processing steps applied separately for each cancer type. Probes listed as potentially noisy by Naeem et al. [17] were discarded, and only probes mapping to autosomal and sex chromosomes were kept. In addition, we discarded probes with *>* 5% of missing values. The remaining missing values were imputed using a k-nearest neighbours approach with the impute package in R (k = 10, rowmax = 0.25) [18]. This resulted in around 277,000 features (probes) per sample (this number varied between cancer types as processing was applied separately to each one). As M-values are more homoscedastic than beta-values [19], we transformed beta-values to M-values using the function shown in Equation (1).

(1) $$M=\log_{2}(\frac{\beta }{1-\beta })$$

For the multiclass data, features were obtained by taking the intersection of features of every cancer type, and the normal class was obtained by pooling all the normal samples from different tissue types together. When pre-processing non-TCGA data sets, where the non-normalized data were available, we computed the beta values from methylated and unmethylated counts (to avoid issues with different normalization methods) and selected the same features as the previously processed TCGA data. Many data sets contained NA values, and unlike before, where we relied on the whole data set to impute the values, we set them to a constant beta of 0.5. This simulated a real-world testing scenario, where you might test one sample at a time and not have access to the whole data set of samples. Lastly, these beta values were transformed to M-values.

### Classification models and metrics

Throughout this study, we used both binary and multiclass models. Each binary model compared one tissue type, distinguishing cancer from normal, and the multiclass models utilized all 13 tissue types and normal samples. Note that in the binary models, the normal class was only normal samples for that tissue, whereas in the multiclass models, the normal class was normal samples from all tissue types pooled together. For each model, the input data were split into training and test sets, with 25% of samples in the test sets.

To begin with, we tested two simple classification models: logistic regression and an SVM. Both models were created and tuned using the package sklearn [20] in Python (version 3.7.5). Hyperparameter tuning on the training set using 5-fold cross-validation selected the default values in most cases, except for the binary logistic regression using the Newton solver and the multiclass SVM using gamma = ‘auto’.

An XGBoost model based on gradient boosted decision trees was created, using the XGBoost (https://xgboost.ai/, RRID: SCR 021361) Python package [21]. Hyperparameter tuning for the binary models resulted in 450 estimators with a maximum depth of ten and a learning rate of 0.189. The multiclass model had eight hundred estimators with a max depth of three and the same learning rate. In this model, 50% of features were randomly sampled when constructing each tree and 50% of samples were taken in each iteration, which helped to prevent over-fitting.

Finally, a multiclass feed forward neural network, namely EMethylNET, which is based on the multiclass XGBoost model was produced. XGBoost models assign an importance to each of their input features, and an importance above zero indicates that this feature is helpful for classification. All input features with an importance greater than zero (3,388 features) were used as input for the neural network. Similar to all other models, we trained it on our TCGA training set. We conducted a hyperparameter search using the Python package Talos [22], using 30% of the training data as a validation set for each hyperparameter test. For training, we used the Adam optimizer [23] with cross-entropy loss. A variant of early stopping was used, where the model was trained for a full five hundred epochs, and the model at the epoch with the highest validation set accuracy was taken as the final model. For evaluation, we used standard accuracy, precision and recall metrics. We also report the *F*_1_ score, which is the harmonic mean of precision and recall, as shown in Equation (2).

(2) $$F1=2\cdot \;\frac{\left(\mathrm{precision}\;\cdot \;\mathrm{recall}\right)}{\left(\mathrm{precision}\;+\;\mathrm{recall}\right)}$$

In addition, we report the Matthews correlation coefficient (MCC) measure (see Equation (3)), as it portrays a more comprehensive measure of performance, especially with imbalanced classes in the binary case [24].

(3) $$\mathrm{MCC}=\frac{\left(\mathrm{TP}\cdot \mathrm{TN}-\mathrm{FP}\cdot \mathrm{FN}\right)}{\left(\mathrm{TP}+\mathrm{FP}\right)\left(\mathrm{TP}+\mathrm{FN}\right)\left(\mathrm{TN}+\mathrm{FP}\right)\left(\mathrm{TN}+\mathrm{FN}\right)}$$

We also report the area under the curve (AUC) for both receiver operating characteristic (ROC) curves and precision–recall curves. For both metrics, one is a perfect score and 0.5 is the score from a random model (assuming classes are balanced). For the multiclass AUCs, an AUC was generated for each class using the one-vs-all strategy, which reflects the model’s ability to distinguish each class from the rest of the classes.

### Biological feature interpretability

#### Multiclass PCC importance analysis

Importances of the multiclass probes contributing to classification (PCCs) were obtained from the trained XGBoost model, which used the gain measure as the feature importance.

#### SHAP values

The shap package in Python was used for analysing SHAP values of the multiclass DNN [25]. A stratified sample of 10% of the training set was used as the background set and a stratified sample of 10% of the whole data set (training and test) was used to calculate the SHAP values.

#### Probe annotation and mapping

Probes with an XGBoost importance score *>* 0 were mapped to genes that were overlapping or that had overlapping promoter regions (taken as the 1500 base pair window upstream of the transcription start site). Each probe was mapped to all genes that fulfilled this property. Then, we went through the multiclass probe list manually and refined probes that mapped to multiple genes, removing mapped genes where it was obvious that the gene was not being affected by the probe. This process removed 161 genes from the multiclass gene list. The gene annotation data were obtained from Ensembl (version 101) using the R package biomaRt (https://bioconductor.org/packages/biomaRt/, RRID: SCR 019214) [26, 27], and the mapping functionality was implemented using the R package, ChIPpeakAnno (http://www.bioconductor.org/packages/release/bioc/html/ChIPpeakAnno.html, RRID: SCR 012828) [28].

#### Differential methylation analysis

Differential methylation analysis was performed using the R package TCGAbiolinks [15], and the input data were M-values of the probes after filtering (see Data pre-processing). Differentially methylated probes were found by the Wilcoxon test using the Benjamini-Hochberg false discovery rate adjustment method. The probes with an adjusted *P*-value *<* .01 and an absolute mean difference of above 2 were selected.

#### Enrichment analysis

##### Gene ontology over-representation analysis

Functional enrichment analysis was carried out using the R package gprofiler2 (https://biit.cs.ut.ee/gprofiler/page/r, RRID: SCR 018190) [29] with the Bonferroni correction method. The background set were the XGBoost input probes (ie, the microarray probe list after filtering) mapped to genes. This result was then visualized by REVIGO (http://revigo.irb.hr/, RRID: SCR 005825) [30] using the settings: small, Homo sapiens GO terms, SimRel similarity. The scatter plot in Fig. 5a was based on the R script provided by REVIGO, and the visible labels are the twenty most significant terms with four or more parents in the GO Biological Process hierarchy (to avoid very general terms).

##### Gene set over-representation analysis

Fisher’s exact tests were performed on two cancer gene sets: COSMIC Cancer Gene Census [31] (https://cancer.sanger.ac.uk/census, RRID: SCR 002260), OncoKB (https://www.oncokb.org/, RRID: SCR 014782) Cancer Gene List [32], and the TF Checkpoint 2.0 resource (https://www.tfcheckpoint.org,RRID: SCR 023880) to determine overlap with translational regulators to assess the overlap with the multiclass gene list. In these analyses, we only included genes present in our background gene set, i.e. the microarray probe list (after filtering) mapped to genes.

#### Text mining

The Pangaea package [33] was used for text mining of over four million cancer-related PubMed (https://pubmed.ncbi.nlm.nih.gov/, RRID: SCR 004846) abstracts (downloaded in 2020) that were associated with cancer. We analysed the abstracts that referred to at least one of our multiclass genes, which was a total of 183,909 abstracts. The output of Pangaea is available as an excel spreadsheet in supplementary data (Supplementary File 1).

#### Pathway enrichment analysis and visualization

KEGGprofile [34] was used for gene set enrichment of KEGG pathways for the multiclass gene list. Transformation of the Ensembl IDs to Entrez gene IDs was required (losing some unmappable genes in the process), and the background set was the microarray probe list (after filtering) mapped to genes. For visualization, KEGG pathways were retrieved using the R package KEGGgraph (https://bioconductor.org/packages/KEGGgraph/, RRID: SCR 023788) [35] and KEGG IDs were converted to Ensembl IDs using the R package biomaRt [26, 27]. Pathways were visualized with the NetworkX Python package (https://networkx.org/, RRID: SCR 016864) [36], and only multiclass genes were shown. For each multiclass gene, the difference in average methylation between cancer and normal is displayed as the node colour. More specifically, for each cancer type and each PCC, the mean M-value of the cancer samples minus the mean M-value of the normal samples for that cancer type was taken. Where multiple PCCs mapped to the same gene, the PCC with the maximum absolute difference was taken.

For the visualization of the pathway network, sixty cancer-related KEGG pathways were collected. Only pathways with more than three multiclass genes were kept (resulting in fifty-six pathways). Interaction data were collected for all multiclass genes, from STRING (http://string.embl.de/, RRID: SCR 005223) [37] (using all interactions from the default 0.4 confidence), GeneMania (http://genemania.org/, RRID: SCR 005709) [38] (with all data sources selected) and GeneWalk (https://github.com/churchmanlab/genewalk, RRID: SCR 023787) [39]. These pathways and interaction data were visualized as a network with Cytoscape software (http://cytoscape.org, RRID: SCR 003032). Each node represented a pathway, and the multiclass genes in that pathway were visualized as smaller shapes around the nodes. The interaction data was summarized into pathway interactions—if a gene in one pathway interacted with another gene in a different pathway, an edge was drawn between those two pathways. In addition, data from the COSMIC Cancer Gene Census, version 93 [31], were integrated.

##### Pan-cancer methylome network model

A model of the pan-cancer methylome network incorporating Molecular Mechanisms of Cancer pathway from the Ingenuity Pathway Analysis (IPA) resource (http://www.ingenuity.com/, RRID: SCR 008653) [40] and the *Pathways in Cancer (Human)* pathway from the KEGG pathway database (https://www.kegg.jp/kegg/pathway.html, RRID: SCR 012773) [41, 42] was produced using PathVisio software (https://pathvisio.org/, RRID: SCR 023789)[43]. Multiclass methylation features mapped to genes were displayed as blue nodes (non-coding genes highlighted in yellow), or purple if they were also known cancer genes from Cosmic Cancer Gene Census or OncoKB. Interaction between nodes is derived from the literature, pathway databases (including IPA and KEGG) and protein-protein interaction data sets (STRING). The model was produced as a gpml object, adhering to the Systems Biology Graphical Notation (SBGN) standard. Direct interactions are shown as complete black lines and indirect interactions as broken black lines, respectively. Catalytic interactions are shown as red edges, inhibitory interactions as blue edges, and protein–protein interactions as orange edges between nodes.

#### Long non-coding RNA analysis

The gene type annotation data were obtained from Ensembl (version 101). Literature evidence was obtained using the Pangaea tool [33], where cancer hallmark keywords were extracted from the abstracts that mentioned at least one of the multiclass lncRNAs. Additionally, we used two cancer lncRNA databases, Lnc2Cancer 3.0 (http://bio-bigdata.hrbmu.edu.cn/lnc2cancer/, RRID: SCR 023781) [44] and CRlncRNA [45]. For Lnc2Cancer, we searched for cancer hallmark keywords in the description column, and for CRlncRNA, these cancer hallmark keywords were included explicitly. LncRNAs found in one or more of these two sources were plotted in a heatmap showing the average methylation (beta value) for all BRCA samples. The differential expression (log_2_ fold change) was obtained using the DESeq2 package (https://bioconductor.org/packages/release/bioc/html/DESeq2.html, RRID: SCR 015687) [46].

##### Comparison with cancer lncRNAs

We compared our lncRNAs to the Cancer LncRNA Census (CLC) [47], using a Fisher’s exact test. We then carried out a pared-down version of their CLC features analysis, following a method as similar as possible. In each of these tests, a Fisher’s exact test was used when not otherwise specified. Gene location and length data were obtained from Ensembl (version 101) using the R package biomaRt [26, 27].

*Close to cancer-associated and non-cancer-associated germline SNPs.* Data were obtained from the GWAS Catalog (NHGRI-EBI’s catalog of published genome-wide association studies (http://www.ebi.ac.uk/gwas, RRID: SCR 012745) [48]. Cancer SNPs were found using keywords ‘cancer,’ ‘tumor,’ ‘tumour,’ and non-cancer SNPs were all other SNPs. We tested whether the closest cancer/non-cancer SNPs to the lncRNAs were within a distance threshold (1 kb, 10 kb, and 100 kb were tested).

*Within 1 kb of the COSMIC cancer gene census genes.* For each background and multiclass lncRNA, the distance to the closest COSMIC cancer gene [31] was computed, and we tested whether that distance was under 1 kb more (or less) frequently for the multiclass lncRNAs.

*Epigenetically silenced in tumours.* The multiclass lncRNAs were tested against a list of cancer-associated epigenetically silenced lncRNA genes (CAESLGs) [49].

*Differentially expressed.* The multiclass lncRNAs were tested against a list of dysregulated lncRNAs in a range of cancer types (BRCA, COAD, HNSC, KIRC, lung adenocarcinoma (LUAD), lung squamous cell carcinoma (LUSC), and PRAD) [49].

*Gene and exon lengths.* To test the difference in lengths, a Wilcoxon rank sum test was performed over the logged lengths (to ensure equal variance). For the exon length, the longest transcript for each gene was taken.

*Higher expression levels.* TCGA expression data were used, normalized by the TMM method [50] using the edgeR package (http://bioconductor.org/packages/edgeR/, RRID: SCR 012802) [51], and averaged across samples. A Wilcoxon rank sum test over the logged expression values was used to test expression differences.

*Conservation.* Phast 100-way conservation scores were downloaded [52, 53] and all conservation scores that overlap with background, or multiclass lncRNA gene bodies were taken. The mean conservation score per gene was computed, and then the difference in conservation between multiclass and background lncRNAs was tested using a Wilcoxon rank sum test.

#### Survival analysis

To determine whether the gene sets could differentiate survival, the following was executed for each gene list from the binary XGBoost models. TCGA expression data were normalized (using the variance-stabilizing transformation in the DESeq2 package [46]), and matched TCGA survival data were obtained. A Cox proportional hazards regression model (using the R package survival (https://CRAN.R-project.org/package=survival, RRID: SCR 021137) [54], version 3.1.8) was fitted on each gene separately along with the age, stage, and gender as covariates (excluding gender for BRCA, PRAD and UCEC). The genes that had a significant effect on survival (using the Wald statistic *P*-value with a .05 cutoff) were selected and a Cox proportional hazards regression model was fitted over all these selected genes. Kaplan–Meier curves were computed (using the survival package) by splitting the samples into a high and low hazard, split by the median hazard.

Then a similar analysis that splits the samples up into a train and test set was run, to show whether the gene lists could predict survival. This was repeated thirty times to get a distribution over the test set performance. After gene normalization, samples were split into stratified train (75%) and test (25%) sets. Using only the train set, a Cox proportional hazards regression model was fitted on each gene separately and selected genes as before. Then three Cox proportional hazards regression models were fitted to the train set—one using just the selected genes, one using just the covariates, and one using the selected genes and covariates. We then used these models to predict the hazard on the test set, and plotted time dependent ROC curves (using the R package timeROC [55], version 0.4) on these predicted hazards.

## Results

### Overview

We utilized machine learning approaches to identify cancer-specific changes from normal tissue-specific methylation. DNA methylation microarray data from 13 cancer types and corresponding normal tissues were utilized. Illumina Infinium array-based methylome data were used in this study and data were extracted, cleaned, and processed as described in the Methods. Analysis of this methylation microarray data identifies the ratio of the methylated probe intensity over the overall intensity, known as the beta value, at given CpG locations using a pair of methylated and unmethylated probes.

**Figure 1.**
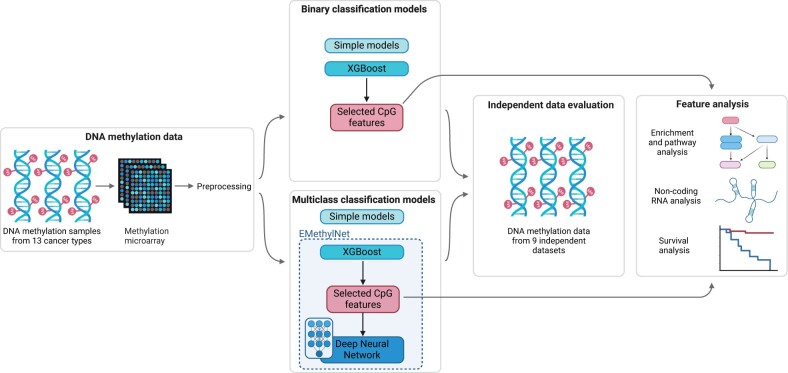
Overview of method. DNA methylation microarray data from 13 cancer types and corresponding normal tissues were collected from TCGA and preprocessed. For binary and multiclass classification tasks, three types of models were trained: Simple models (logistic regression and support vector machines), XGBoost, and EMethylNET, a model consisting of XGBoost combined with a Deep Neural Network. Then the models were evaluated on independent data sets and an analysis of their features used in classification was performed

In this study, we trained and evaluated four different model types: logistic regression, support vector machines (SVM), gradient boosted decision trees (XGBoost), and a deep neural network (DNN). See Fig. 1 for a visual overview. For the first three model types, both binary and multiclass classification models were created.

### Cancer types can be accurately classified using both binary and multiclass methods

Here, we present the results from the XGBoost and DNN models. We also trained and tested SVM and logistic regression models. In addition to measures of accuracy such as ROC AUC and F_1_ score, we also provide the MCC measure. MCC is used to portray performance of the binary classification models and is especially useful when class imbalances are present. The SVM models did not outperform the XGBoost or DNN models: the SVM binary models reached an average MCC of 0.894 and the SVM multiclass model reached an MCC of 0.956. The binary logistic regression models had an average MCC of 0.960, outperforming the binary XGBoost models on average (average MCC of 0.919); however, their performance varies across cancer types: logistic regression models perform better for 5 cancer types, XGBoost models perform better for 4 cancer types, and both achieve the same MCC for four cancer types. The multiclass logistic regression model (MCC score of 0.973) did not outperform the multiclass XGBoost or DNN (MCC scores of 0.980 and 0.976). Since the binary logistic regression models did not substantially outperform the binary XGBoost models and the multiclass logistic regression achieved a lower MCC score than the multiclass XGBoost and DNN, we focus our analysis on the XGBoost and DNN. For detailed performance metrics of the SVM and logistic regression models, see Supplementary Tables S1 and S2.

#### Detection of the cancer states through binary classification of DNA methylation from individual tumour and normal tissues

**Figure 2.**
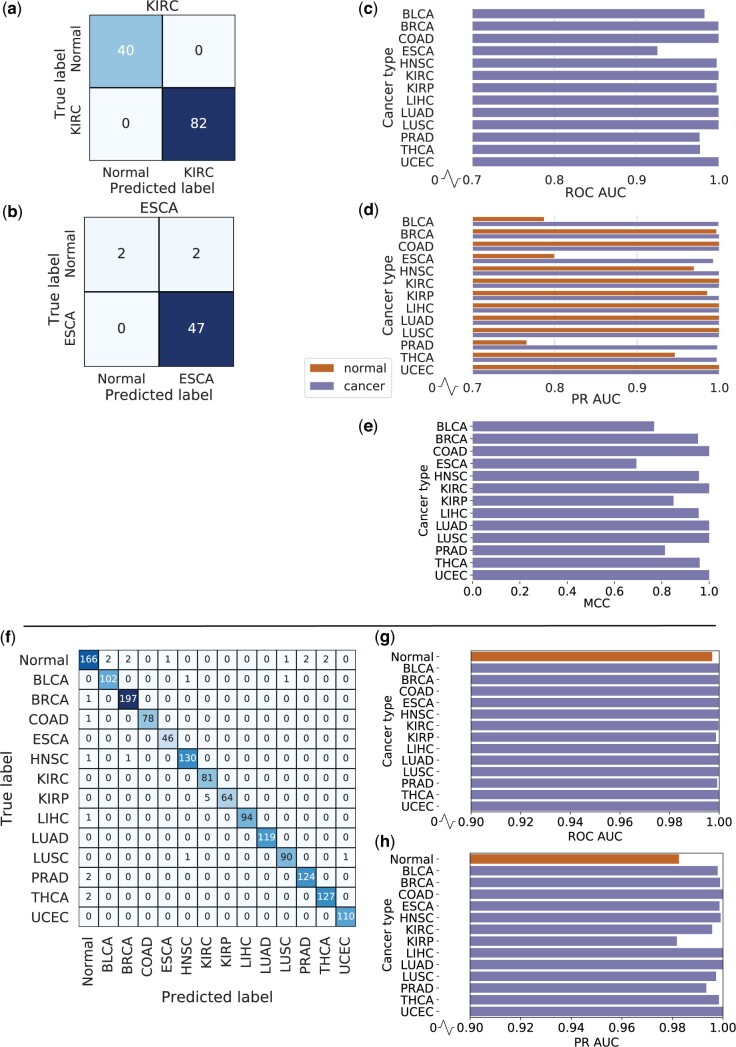
Performance of the binary and multiclass XGBoost models on the TCGA test set. **a** and **b** Confusion matrices of the best (KIRC) and worst (ESCA) performing binary XGBoost models. **c** AUC of the ROC curves for all binary XGBoost models. **d** AUC of the Precision Recall (PR) curves for both cancer and normal classes of all binary XGBoost models. Note that the scales of **c** and **d** start from 0.7. **e** MCC scores for all binary XGBoost models. **f** shows the confusion matrix, **g** shows the AUC of the ROC curves for each class, and **h** shows the AUC of the Precision Recall (PR) curves for each class of the multiclass XGBoost model. Note that the scales of **g** and **h** start from 0.9

XGBoost, a type of gradient boosted tree model, is an iterative ensemble machine learning approach [21]. We trained 13 binary XGBoost models, one for each cancer type. DNA methylome data from TCGA were used in training and testing the models, with a total of 6224 samples. Each model learns to classify between cancer and normal samples (adjacent matched normal tissue) for its tissue type. Overall, there was good performance on the test set, with five out of 13 models achieving a perfect test set performance (COAD, KIRC, LUAD, LUSC, and uterine corpus endometrial carcinoma [UCEC]). Across all models, the average accuracy was 0.987 and the average MCC (a performance measure unaffected by severe class imbalance) was 0.919, demonstrating that the models can accurately classify cancer and normal samples. Figure 2a–e shows the confusion matrices for the best and worst performing models, AUC of ROC curves and precision-recall curves, and the MCC scores for all models. Performance metrics for all binary models can be found in Supplementary Tables S3.1. A key issue with these binary models is the major class imbalance. The average fraction of normal samples is 0.135 (see Supplementary Table S12 for the numbers of normal and cancer samples for each tissue type), which reveals why the average MCC is considerably lower than the average accuracy. In addition, the lowest performing model, ESCA, with an accuracy of 0.961 and MCC of 0.693, is the tissue type with the lowest number of normal samples, of which there were only sixteen. This sparsity of data contributed to its worse performance.

#### The multiclass classification of 13 cancer and normal tissues is more robust

Here, we trained a single multiclass XGBoost model on the whole of the training data. There were classes for each of the 13 cancer types and a single normal class, which contained normal samples from every cancer type. The model was now required to learn the differences between 13 tissue types in addition to the differences between cancer and normal tissue samples, making it a more challenging task than the previous binary classification. However, there was no longer a large class imbalance due to pooling of the normal samples together. As shown in Fig. 2f–h, the performance of the test set was very good for all classes. The model can discriminate each of the 13 cancer types and normal samples with a high degree of accuracy. The overall accuracy was 0.982 and the overall MCC was 0.980, see Supplementary Table S3.2 for the detailed metrics.

### Models achieve high accuracy on independent heterogeneous data sets

To determine the robustness of our models, we evaluated our XGBoost models on several independent data sets representing different cancer types, amounting to a total of 940 samples. These were more heterogeneous than the TCGA data used for training. Two data sets included some adenoma samples (COAD and THCA), one data set consisted of samples from early-stage tumours, some of which were later shown to recur (LIHC), and one data set included some Human papillomavirus (HPV) positive samples (HNSC). The data sets also came from seven different countries, viz., Iceland (BRCA), USA (COAD), Australia (ESCA), UK (HNSC and ESCA), China (LIHC and KIRC), Canada (PRAD), and Brazil (THCA).

#### Binary models show good performance on independent data sets

**Figure 3.**
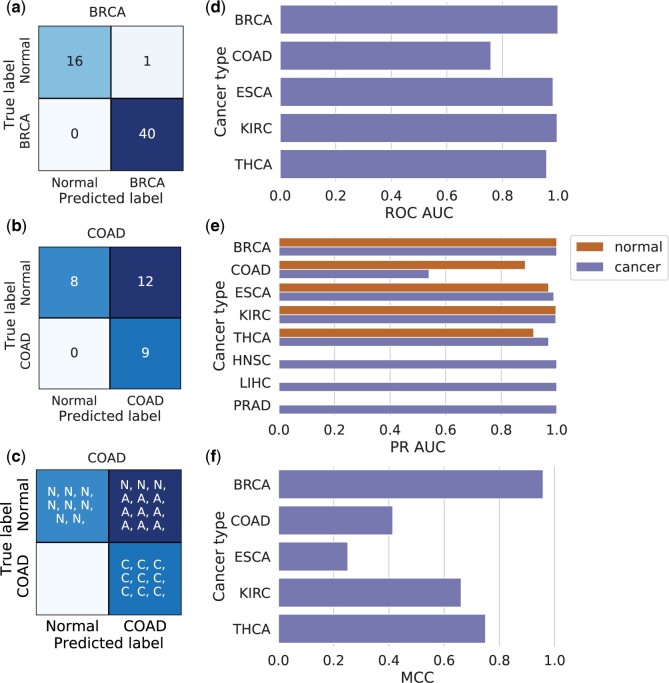
Performance of the binary XGBoost models on independent data sets. **a** and **b** Confusion matrices of the best (BRCA) and worst (COAD) performing binary XGBoost models (according to the ROC AUC scores) on the independent data sets. **c** Detailed confusion matrix for COAD showing the predictions of Normal (N), Adenoma (A), and Cancer (C) samples. **d** AUC of the ROC curves for binary XGBoost models where the independent data set included normal samples. **e** AUC of the Precision Recall (PR) curves for both cancer and normal (where available) classes of binary XGBoost models on the independent data sets. **f** MCC scores for binary XGBoost models where the independent data set included normal samples. For **d**, **e** and **f**, ESCA is the average of the two ESCA independent data sets

When these independent data sets were tested, most of the binary XGBoost models (trained on TCGA data) performed well, illustrated by Fig. 3. Confusion matrices of the best and worst performing binary XGBoost models are shown in Fig. 3a and b. In terms of ROC AUC (Fig. 3d), the highest performing model was the BRCA model, with a perfect ROC AUC of 1.0, and the lowest performing was the COAD model, with a ROC AUC of 0.758. The precision–recall AUC results show similar trends (Fig. 3e). In terms of MCC (Fig. 3f), the lowest performing model was the ESCA model, which is expected given the major class imbalance in the ESCA training data set.

Regarding the COAD model, its confusion matrix in Fig. 3b shows that it predicted 12 normal samples as cancer. Nine out of these twelve samples are in fact adenomas; benign tumours of glandular origin (see Supplementary Table S4 for the number of normal, adenoma and cancer samples in the COAD independent data set). A confusion matrix that also shows whether the samples are Normal (N), Adenomas (A), or Carcinomas (C) is shown in Fig. 3c, illustrating that all adenomas are classified as cancer. This was unexpected, as there were no adenomas in the training data set, and instead of randomly classifying them, the model found some cancer-associated signal in adenoma samples in the independent data set.

A similar trend was identified in the other independent data set with adenomas. In the THCA model, eleven out of 17 adenomas were predicted to be cancer (see Supplementary Table S4 for the number of normal, adenoma and cancer samples in the THCA independent data set). In detail, all occurrences of ‘follicular adenoma’, and ‘follicular adenoma/Hürthle cell’ were classified as cancer (n = 8), all ‘lymphocytic thyroiditis’ were classified as normal (*n *=* *3), and ‘nodular goitre’ was split evenly between the two classes (n = 6).

#### EMethylNET, a model consisting of a DNN model trained on features learnt from multiclass XGBoost, improves performance

**Figure 4.**
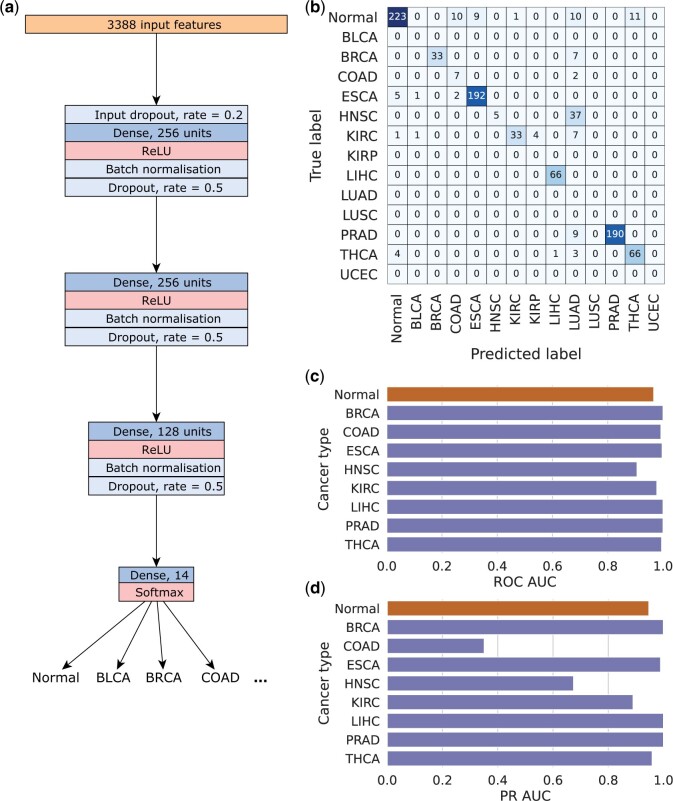
Architecture of the feed forward neural network (**a**) and its performance on all independent data sets. **b** shows the confusion matrix, **c** shows the AUC of the ROC curves for each class, and **d** shows the AUC of the Precision Recall (PR) curves for each class. The two ESCA data sets are combined into one ESCA class. The colour orange denotes normal and purple denotes cancer. Note that we do not have independent data sets for every cancer type (the independent data sets used lacked BLCA, KIRP, LUAD, LUSC and UCEC samples). Nevertheless, for the confusion matrix in **b** all 14 classes are retained in the rows to maintain a square configuration, enhancing readability

The results for the multiclass XGBoost model on the independent data, which had an accuracy of 0.68 and MCC of 0.661, can be found in Supplementary Table S6. With the aim of creating a more robust model and improving these results, we designed EMethylNET, a feed-forward neural network based on our XGBoost model, as shown in Fig. 4a. The input features of EMethylNET were the features the multiclass XGBoost model learnt to utilize for classification, referred to as the probes contributing to classification, see below. See Supplementary Table S5 for EMethylNET’s results on the TCGA test set. The results on the independent data sets, which had an accuracy of 0.867 and MCC of 0.844, are shown as a confusion matrix (Figure 4b), AUC of ROC (Figure 4c) and AUC of PR (Figure 4d) and in Supplementary Table S7. The only data set that did not reach a *F*_1_ score of at least 0.8 (excluding COAD, as it contains adenomas, see above) was HNSC.

#### Comparison of EMethylNET with related cancer classification studies

The detection and classification of cancer using methylation-based approaches is a large and growing body of literature. A diverse range of approaches and objectives have been investigated, from binary classification of cancer using tissue data [56], to multiclass classification using data from liquid biopsies [57]. Here, we conduct a comparative analysis of EMethylNET with other related works that utilize machine learning for pan-cancer multiclass classification of DNA methylation data from tissue samples. These related works [58–69] are listed in Table 1. Various machine learning approaches have been used, from logistic regression to DNNs, with tree-based methods (random forest and XGBoost) being a popular approach (6/12 works).

First, we provide a performance comparison of EMethylNET to these related works. We only compare works that provide test set scores on TCGA (we do not attempt to run their models). This comparison is not exact, and it is important to note the following shortcomings: the test sets contain different samples and have different sizes, the related works have slightly different classification tasks (for example, some only consider cancer samples, some define a normal class for each tissue type and some have separate classes for metastatic samples) and some classes in related works are not comparable with our classes (for example, some works combine cancer types found in the same tissue type). In addition, we can only compare with the metrics reported in the publication, and so different metrics are compared for different works.

First, we compare with Hao 2017 [58]. They classify four cancer types and four normal tissues, and so we can only compare with the four cancer types (as our normal samples are pooled). Supplementary Table S8 shows the precision and recall metrics, indicating that EMethylNET achieves comparable performance for these cancer types (higher precision for COAD, LIHC and LUAD, and higher recall for LUAD). Next, we compared with Ibrahim 2022 [67]. They perform a slightly different task, as they do not include a normal class, and they combine colon and rectal tumour data sets, so we cannot compare performance on COAD. Supplementary Table S9 shows the ROC AUC scores, showing that EMethylNET achieves the same ROC AUC or higher in all classes (when rounding to three decimal places). Ibrahim 2022 also externally validate their model on the independent BRCA (GSE52865) and THCA (GSE97466) data sets. Again, this is not a direct comparison because the rest of their independent external validation data set differs from ours (which affects the one-vs-all approach to calculating AUC scores). For the BRCA data set, they report a ROC AUC of 0.928, and we achieve a ROC AUC of 0.99997. For the THCA data set, they report a ROC AUC of 0.990, and we achieve 0.99463. Next, we compare with Zheng 2020 [63]. They do not include normal samples, and they classify the cancer origin site, which again is a slightly different task to ours. We cannot compare KIRC, kidney renal papillary cell carcinoma (KIRP), LUAD and LUSC classes as they combine them into generic kidney and lung classes. The ROC AUC, precision and recall metrics are shown in Supplementary Table S10, indicating that we achieve comparable performance. EMethylNET’s ROC AUCs are the same or higher in all classes (when rounding to two decimal places), precision is higher in 4/8 classes and recall is the same or higher in 5/8 classes. Lastly, we compared with Modhurkur 2021 [66]. As they have distinct classes for each metastatic cancer and each normal tissue type, they address a more challenging task. Supplementary Table S11 shows the precision, recall and F1 metrics, which shows that we achieve comparable performance. EMethylNET’s precision is the same or higher in 6/13 classes, recall is the same or higher in 10/13 classes, and F1 is higher in 8/13 classes. In summary, we have shown that EMethylNET achieves competitive test set performance amongst comparable works.

### Biological feature interpretability

A key advantage of using an interpretable method such as XGBoost is that the features utilized for classification can be identified. In our case, these were the CpG probes with a feature importance of above zero, which we refer to as PCCs. Surprisingly, most PCCs from the binary models were found not to be differentially methylated in each respective cancer type. Only 65/221, 56/318 and 29/179 PCCs from the BRCA, PRAD, and THCA binary models, respectively, were found to be differentially methylated, as shown in Supplementary Fig. S1.

We explored the PCCs from the multiclass XGBoost model (exactly the input features to EMethylNET). The importance scores of the most important PCCs are shown in Supplementary Fig. S2a, which shows that most of the importance is captured by the top one hundred PCCs. The most important PCC, cg16508600, is at position chr1:204562255 and does not map to any gene. The closest gene is RNA5SP74, which is *∼*200bp away, and the location of this PCC coincides with a C *>* T SNV (rs567580996). The second most important, cg14789818, is ∼200bp upstream of RNA5SP77 on chromosome 1. The third most important, cg03988778, is near the promoter of SVIP and AC006299.1. Supplementary Fig. S2b shows the distribution of methylation of the top ten important probes, indicating that they commonly differentiate one class from the rest. For example, the most important PCC differentiates BRCA from all other classes, and the third most important differentiates a sizable proportion of HNSC class.

An interpretation of the multiclass DNN model can be achieved by analysing its SHAP (SHapley Additive exPlanation) values [25]. The feature with the highest average impact on model output (the highest average absolute SHAP value) is cg15267232, which is within GATA3, and the feature with the second-highest average impact is cg22455450, which is within ZNF808. The feature with the third-highest average impact is cg22541735, which is within HOXD9 and HOXD-AS2. Interestingly, the feature with the 10th highest average impact (cg14789818) is also the second most important feature for the multiclass XGBoost model. Supplementary Fig. S3 visualizes the features with the highest average absolute SHAP values.

#### The proximal genes of the multiclass model’s features are enriched in genes contributing to hallmarks of cancer, carcinogenesis, and transcriptional regulation

The PCCs can be mapped to the proximal genes—genes where the gene body or promoter region (taken as the 1500 base pair window upstream of the transcription start site) overlap the PCCs. We will refer to the genes obtained by mapping the multiclass PCCs to proximal genes as ‘multiclass genes’.

**Figure 5:**
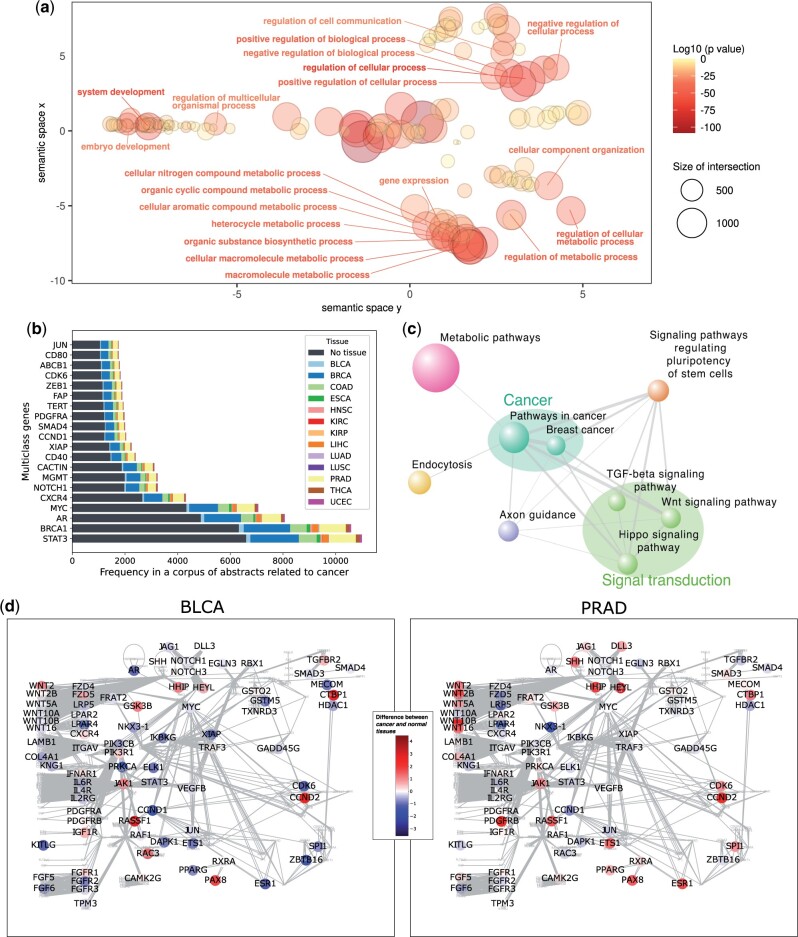
Cancer processes, genes, and pathways in the multiclass gene list. **a** A REVIGO visualization showing the significant Gene Ontology terms, restricted to the biological process domain. Only a small selection of terms is labelled. **b** The 20 multiclass genes found most often in abstracts about cancer. Colour indicates the number of abstracts also specifying a tissue. **c** A visualization of the significant KEGG pathways, where the size of the node (pathway) is the amount of overlap between the multiclass gene list and the pathway, and the width of the edge indicates the amount of overlap between the two pathways. **d** The Pathways in cancer KEGG pathway, showing only multiclass genes. Each multiclass gene is coloured by the difference in average methylation between cancer and normal for two cancer types: BLCA and PRAD

We performed functional enrichment analysis on the multiclass genes. A visualization of the significant Gene Ontology terms, restricted to the Biological Process ontology, is shown in Fig. 5a. This shows that our multiclass gene list is enriched in development, regulation of signaling, processes involved in gene expression changes, and the regulation of a wide variety of metabolic processes. Over-representation analysis revealed that there is significant overlap between the multiclass genes and the COSMIC Cancer Gene Census [31], with an overlap of 140 genes (19.0% of COSMIC genes) (Fisher’s exact test, *p *=* *8.7e *−* 17). We also found significant overlap between the multiclass genes and the OncoKB Cancer Gene List [32], namely 217 genes (19.7% of OncoKB cancer genes) (Fisher’s exact test, *p *=* *4.5e *−* 27). Furthermore, analysis of multiclass features using the TF checkpoint 2.0 database indicated that 17.2% (546 genes) (Fisher’s exact test, *p *=* *2.4e *−* 39) are also transcriptional regulators.

We also looked at the overlap with established DNA methylation biomarkers in cancer, by comparing with the genes used by commercially available DNA methylation-based biomarker assays [70]. Out of the 13 genes measured by these assays, four overlap with our multiclass genes (RASSF1, SEPTIN9, SHOX2, MGMT). During normal expression, RASSF1A represses cell cycle proteins cyclin A2 and cyclin D1, leading to cell cycle arrest and plays a significant role in microtubule stability and modulates apoptosis. Furthermore, RASSF1 inactivation is one of the most common epigenetic changes in cancer [71]. Similarly, SEPTIN9 participates in cytokinesis during the cell cycle [72], while SHOX2 is a transcription factor involved in proliferation, migration and colony formation [73] and MGMT inhibits tumour formation [74]. All these genes are well-known prognostic biomarkers in cancer. There are also several multiclass genes in the same family as these 13 genes (such as NDRG3, BMP8A, OTX2, ONECUT1).

Text mining a corpus of 183,909 PubMed cancer-related abstracts that mention at least one multiclass gene revealed that the cancer literature provides evidence for the multiclass genes. 65.6% (2083) of our multiclass genes are found in at least one cancer-related article abstract from PubMed. See Fig. 5b for the genes most supported by the literature. These include well-studied oncogenes such as STAT3, BRCA1, AR, MYC, CXCR4, NOTCH1, SMAD4, TERT, ZEB1, JUN, amongst others. This analyses also demonstrated that just under 40% of these abstracts are additionally associated with at least one of the 13 tissue types included in the multiclass model. BRCA, PRAD and COAD are most commonly found, due to their high prevalence. Supplementary File 1 details the evidence for the multiclass genes in these PubMed cancer-related abstracts.

#### The multiclass genes are enriched in cancer-related pathways and networks

Pathway enrichment analysis using the KEGG pathway database revealed enrichment of pathways related to general cancer hallmarks, such as *Pathways in cancer* (adjusted *P*-value = 4.3e *−* 4), *Metabolic pathways* (adjusted *P*-value = .0214), and signal transduction pathways such as the *Wnt signalling pathway* (adjusted *P*-value = 8.2e *−* 3), *TGF beta signalling*, *Hippo signalling*, *Axonal guidance pathway involved in invasion and metastasis*, and many metabolic pathways. See Fig. 5c for a visualization of these enriched pathways.

The multiclass genes in these pathways displayed different methylation patterns for different cancer types. A visualization of the Pathways in cancer network from KEGG is shown in Fig. 5d for both bladder urothelial carcinoma (BLCA) and PRAD, and in Supplementary Fig. S4 for all other cancer types. This shows that BLCA, KIRC, KIRP, LIHC, THCA and UCEC are mostly hypomethylated whilst BRCA, COAD, LUAD, LUSC and PRAD are mostly hypermethylated. Supplementary Fig. S5a shows a heatmap of PCCs at least 2-fold differentially methylated and their recurrent mutation status (from COSMIC cancer gene census and TCGA significantly mutated list) is indicated. Similarly, the differential methylation of all PCCs is shown in Supplementary Fig. S5b. In addition to mutations and copy number aberrations, the PCC features identified by our analysis contribute to carcinogenesis in multiple cancers via methylation changes of regulatory elements.

**Figure 6:**
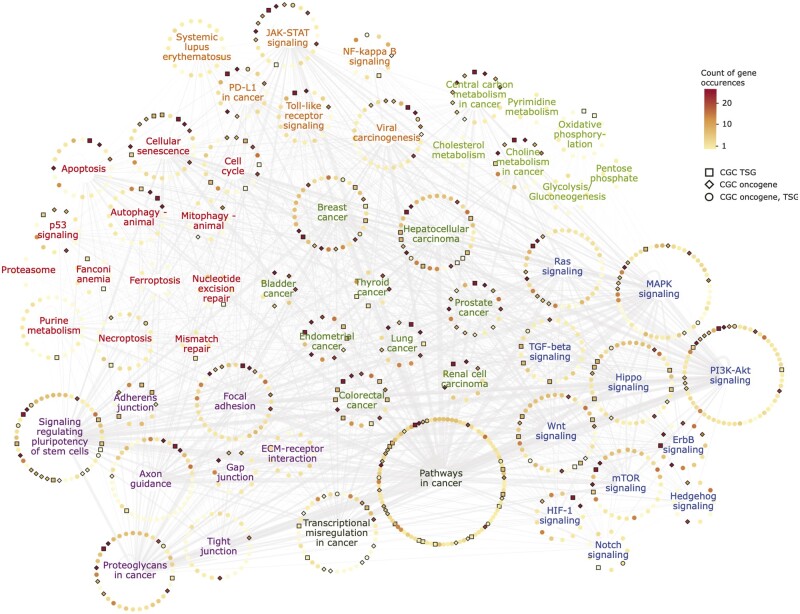
A network of cancer pathways and the multiclass genes. Each circle of nodes is a cancer pathway, and each node represents a multiclass gene. The node colour represents the number of times each multiclass gene is displayed (as they can be in multiple pathways), the edge thickness represents the number of interactions between pathways, and a black outline indicates that the multiclass gene is found in the Cancer Gene Census. The colour of the pathway name represents the pathway category

Furthermore, multiclass genes were found to be present in a broad range of cancer-related pathways, as shown in Fig. 6. These pathways covered a wide range of categories: Individual cancer types, Cell Death and Survival, Tissue Microenvironment, Signalling, Metabolism, and Immune System. This pathway model also shows that many multiclass genes in these pathways are present in the COSMIC Cancer Gene Census [31]. To visualize the multiclass genes in one unified cancer network, we curated a general cancer network, based on two general cancer pathways: KEGG’s *Pathways in cancer* and Ingenuity Pathway Analysis (IPA) *Molecular Mechanisms of Cancer* (both of which our multiclass genes are enriched in, Fisher’s exact test respective adjusted *P*-values = 4.206e *−* 10 and 1.065e *−* 05). This network model is shown in Supplementary Fig. S6 and demonstrates that the multiclass genes span all areas of cellular networks underlying carcinogenesis.

#### Multiclass long non-coding RNAs are associated with oncogenic properties

**Figure 7:**
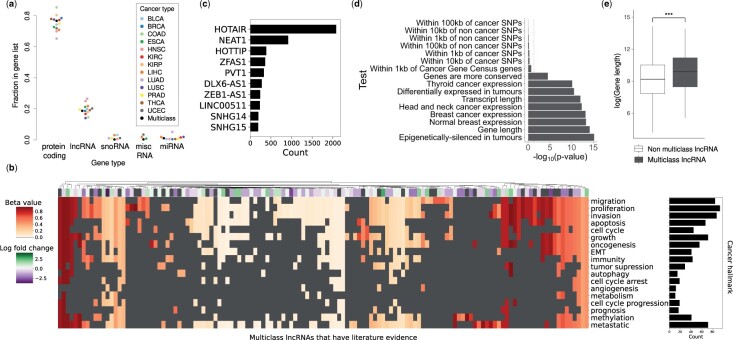
Analysis of the lncRNAs found in the gene lists. **a** The fractions of different gene types in all cancer gene lists, including the multiclass gene list. **b** A heatmap of BRCA data showing the average beta value of the multiclass lncRNAs with literature evidence, and the cancer hallmarks they are associated with. The row annotation indicates the log fold change from differential expression analysis, where non-significant fold change (adjusted *P*-value > 0.05) is in grey. **c** The top 10 multiclass lncRNAs that had the most literature evidence. **d** The significance levels resulting from testing the multiclass lncRNAs for previously observed cancer lncRNA features [41]. The dashed red line indicates the *P*-value = .05 level of significance. **e** Boxplot of the log_e_ gene length of non-multiclass lncRNAs and multiclass lncRNAs. ‘***’ indicates *P*-value < .001

Additionally, we investigated the proportion of protein-coding versus non-coding genes in our gene lists. This is visualized in Fig. 7a for all individual cancer gene lists and the multiclass genes. It shows that, as expected, most genes are protein-coding (around 65% to 85%). However, the proportion of long non-coding RNA (lncRNA) is surprisingly high for all gene lists (around 14% to 26%), which motivated further analysis. We validated some of the multiclass lncRNAs with literature evidence using Pangaea [33] and two cancer lncRNA databases (Lnc2Cancer 3.0) [44] and CRlncRNA [45]. We found evidence for 142 multiclass lncRNAs (out of a total 596 multiclass lncRNAs). See the heatmap in Fig. 7b (and Supplementary Fig. S7a for a larger version), which shows that there is a wide range of methylation values for these lncRNAs, and a range of cancer hallmarks associated with them. The most common hallmarks are proliferation, invasion, and migration. The lncRNAs with the most evidence include HOTAIR, NEAT1, and HOTTIP, as seen in Fig. 7c.

We also compared the multiclass lncRNAs to a set of validated cancer lncRNAs and found that they share some of the same properties. Carlevaro-Fita et al. introduced [47] and Vancura et al. updated the Cancer LncRNA Census 2 (CLC2), which is a list of 492 lncRNAs that have been causally associated with cancer [75]. Our multiclass lncRNAs do have a significant overlap with the CLC2 (Fisher’s exact test, *P*-value = 3.0e − 18); however, this is only 74 overlapping lncRNAs. Carlevaro-Fita et al. also uncovered the properties of genes in the CLC, such as smaller distances to cancer SNPs, higher conservation, and longer gene lengths. By carrying out the same tests on our multiclass lncRNAs, we found that the multiclass lncRNAs share some of these CLC properties. We tested the distances to cancer-associated and non-cancer SNPs, distances to cancer associated genes, epigenetic silencing in tumours, differential expression, gene and transcript lengths, gene expression levels, and conservation. The *P*-values for each of these tests are shown in Fig. 7d. We found that our multiclass lncRNAs did not share any of the same proximity properties (distances to SNPs and cancer genes) but did share all five remaining properties. See Fig. 7e for a boxplot showing that the multiclass lncRNAs have longer gene lengths, and Supplementary Fig. S7b–g for boxplots of the other relevant tests.

#### Models for some of the cancer types can predict 5-year survival

**Figure 8:**
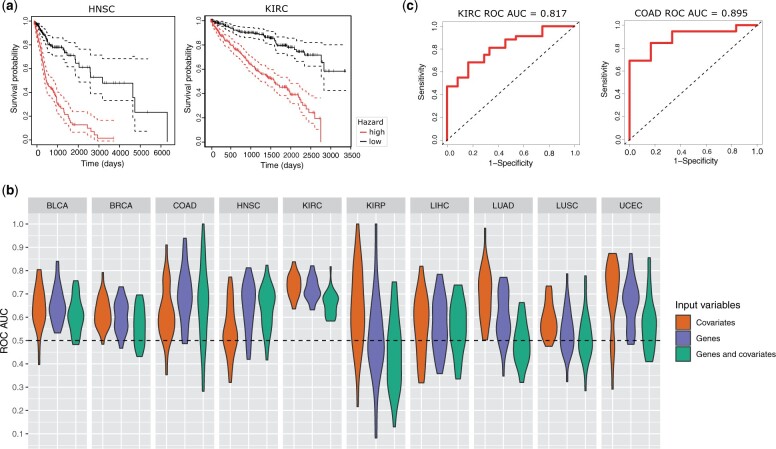
Survival analysis using the gene lists from the binary models. **a** The two most significant Kaplan-Meier curves that differentiate survival: HNSC (*P*-value: 3.15x10-16) and KIRC (*P*-value: 3.06x10-15). **b** The distribution of ROC AUCs when predicting 5-year survival for cancer types with sufficient survival data. Colour represents the three different variations of input variables to the survival models. **c** The best ROC curves for predicting 5-year survival of the cancer types with the highest average ROC AUC: KIRC and COAD

We used the gene lists from the binary XGBoost models to determine whether they could firstly differentiate, and then predict, survival. Survival was computed for each cancer type, using just the expression of the genes from the binary model as input. For every cancer type, the cox proportional hazard model significantly differentiated survival. Figure 8a shows the Kaplan–Meier curves for the most significantly differentiated cancer types, HNSC (*P*-value = 3.15e *−* 16) and KIRC (*P*-value = 3.06e − 15).

Next, we explored whether the gene lists could predict survival on a held-out test-set. The performance differed between cancer types, as shown in Fig. 8b (here we use age, stage, and gender as covariates). This shows that there is a broad distribution for some cancer types (such as KIRP), which could be due to low sample numbers (KIRP has the second-lowest number of samples). Three cancer types did not have enough data to converge—PRAD and THCA both had less than 15 positive samples (events), and ESCA had the least number of samples. However, models for some cancer types could predict 5-year survival consistently well using only genes as input, such as KIRC, COAD, BLCA, HNSC, and UCEC. Figure 8c shows the best ROC curves from the two cancer types with the highest average ROC AUC, KIRC and COAD. This shows their best ROC AUCs are 0.817 and 0.895, respectively.

## Discussion

The early detection of cancer is vital for enabling treatment options that lead to better prognosis. A fundamental requirement for this is to distinguish cancerous from non-cancerous tissue samples accurately. Here, we have utilized epigenetic changes in the DNA methylome and present binary and multiclass machine learning models to classify 13 cancer types and corresponding normal tissues. Our approach achieved good test set performance for all XGBoost models, namely an average accuracy of 0.987 and 0.982 for the binary and multiclass models, respectively. We were then able to show that the PCCs selected by XGBoost can robustly classify cancer when fed into a multiclass deep neural network, namely EMethylNET (accuracy 0.976).

The performance on most independent (non-TCGA) data sets was above a *F*_1_ score of 0.8, and half of the independent data sets achieved an F_1_ score of over 0.9. These independent data sets were more heterogeneous and reflected more realistic situations. Lastly, we demonstrated that multiclass PCCs do have biologically meaningful significance in cancer. Over-representation analysis revealed that the multiclass genes were enriched in processes which are linked to cancer hallmarks, and other cancer and methylation studies report similar Gene Ontology enrichment results [76, 77]. Furthermore, a comprehensive text mining analysis of the literature demonstrates that cancer-associated methylation changes in 892 of our multiclass genes are supported by 7831 publications. We also showed that the multiclass genes set consists of 229 known tumour suppressors and oncogenes, 546 transcriptional regulators and are involved in a wide range of cancer-related pathways and processes. Additionally, we showed that our gene lists contain many non-coding RNA genes, primarily consisting of lncRNAs. This is consistent with a growing body of research showing that lncRNAs and other non-coding RNAs play a key role in carcinogenesis [78–80].

There were two exceptions to the performance of our models, one of them being the independent COAD data set. As indicated in the Results, this low performance can be explained by all adenomas, labelled as normal, being predicted as cancer. Adenomas are dysplastic polyps which can progress via the adenoma–carcinoma sequence to invasive cancer. Therefore, it is common to remove colon adenomas when they are found to stop the possible progression into carcinomas [81, 82] and so this behaviour was inadvertently useful. However, a larger sample size of adenomas would be needed to validate this. The other exception is the HNSC independent data set, which has the lowest performance. HNSC is very heterogeneous, in that it can arise from multiple different tissue sites, and the TCGA HNSC data reflects this. However, the independent HNSC data set only stems from one tissue of origin, the oropharynx, and only 1.55% of the TCGA data stems from the oropharynx. In addition, half of the independent HNSC data set is Human papillomavirus positive (HPV+), which is known to display different methylation patterns [83, 84]. Thus, we were testing on HNSC cancer types with very little TCGA training data, which could explain the poor performance. In addition, the independent HNSC data were often misclassified as LUAD. The uniform manifold approximation and projection visualization in Supplementary Fig. S8 illustrates that out of all TCGA classes, the independent HNSC data were the closest to LUAD. This could be due to a biological reason, such as the independent HNSC data are in fact metastases which originated in the lung, or this could be due to specific data generation or processing artefacts.

We compared EMethylNET with related cancer classification studies and demonstrated similar or better performance against test set data. We also compare these related works with respect to the features selected by the models. The related works all utilized feature selection methods, such as the moderated t-statistic or differential methylation analysis, with multiple works using redundancy filters, for example the Maximum Relevance–Maximum Distance technique [59], and many utilizing multiple feature selection methods in parallel [59, 63, 67, 69, 85]. Thus, most of these approaches start from a highly filtered probe list, and some only use tens of probes in the final classification model (as detailed in Table 1), consequently the models could potentially be biased by the feature selection methods used. In our approach, we did not perform a prior feature selection, but instead let the XGBoost classification model perform the feature selection itself, from an input set of around 277,000 features. For the multiclass case, this resulted in a large set of PCCs, of size 3388, that provided us with an interpretable model and an explainable list of genomic loci for further analysis. Only a handful of the related works have performed feature analysis of the CpGs selected by the model. Ding et al. [62] performed functional analysis of its 7 CpGs and Liu et al. [85] found cancer-related genes near three out of its 12 CpGs. We provide an extensive analysis of our PCCs, encompassing over-representation analyses, extensive literature mining, and pathway enrichment visualizations. Exploring the pan-cancer methylome as a network (Fig. 6 and supplementary Fig. S6) enabled the identification of genes associated with several well-studied cancer-associated pathways, including well-known tumour suppressor and oncogenes present in the collection of our PCCs. These include those genes involved in cancer-associated pathways such as TP53, WNT, Notch, TGF beta/BMP, RAS, MAPK, PI3K-AKT and Hedgehog signalling as well as pathways impacting proliferation, survival and cell death including cell cycle regulators, mitotic checkpoint genes, mitochondrial metabolism, DNA damage responses and apoptosis. In addition, pathways involved in invasion and metastasis-associated processes such as the epithelial mesenchymal transition (EMT)-related genes, axonal guidance pathway, and those involved in adherence junctions and extracellular matrix interactions, Integrin signalling and angiogenesis were present. Furthermore, immune response regulators such as cytokine (IFN, interleukin, chemokine), TLR signalling, and interferon stimulated genes were also present. Finally, genes and pathways affecting global gene expression such as developmental regulators, chromatin remodellers, epigenetic regulators and transcription factors were detected. Investigating these genes in a cancer network context enabled their interactions and relationships to be identified. The pan-cancer methylome also demonstrates that in addition to mutations and genetic aberrations, epigenetic changes have wide-ranging impacts on carcinogenesis. To summarize, in comparison with related studies, we are the first to provide an in-depth feature analysis where the CpGs were selected freely by the model, with no prior feature selection adding potential bias to the feature analysis results.

In conclusion, we demonstrated that XGBoost models are suitable for classifying a multitude of cancer types using only DNA methylation data as input. We additionally designed EMethylNET, a robust deep neural network that was able to generalize to most independent data sets. In addition, we find that mapping the PCCs to genes identifies genes that are enriched in functional properties and pathways linked to carcinogenesis. Depending on the availability of training data, this method can be extended to detect hundreds of cancer types. Future applications include extending this approach to DNA methylation data of cell-free DNA, with the eventual aim being early detection of multiple types of cancer from liquid biopsy approaches. Furthermore, a clear clinical application of this method is screening for specific cancer types or cancers of unknown origin, although the current models are not optimized for this purpose.

## Supplementary Material

bpae028_Supplementary_Data

## Data Availability

The results shown here are in whole or part based upon methylome data generated by the TCGA Research Network: https://www.cancer.gov/tcga. Non TCGA evaluation data sets with the following accession IDs were downloaded from NCBI GEO and ICGC.

## Code availability

The code for this project was produced in a reproducible manner with Python Jupyter and R notebooks, available at: https://github.com/ss-lab-cancerunit/EMethylNET_code. The code and data used to generate most of the figures are also available as a compute capsule https://doi.org/10.24433/CO.1745934.v1 on the CodeOcean computational reproducibility platform enabling the ease of sharing, running and discovery of the code used in this study.
