# Supplementary material for: Early detection and diagnosis of cancer with interpretable machine learning to uncover cancer-specific DNA methylation patterns
Source: Biol Methods Protoc. 2024 Jun 20;9(1):bpae028. doi: 10.1093/biomethods/bpae028 (PMC11186673; doi:10.1093/biomethods/bpae028)
Supplement: bpae028_Supplementary_Data [file bpae028_supplementary_data.zip › EMethylNet_Newsham_etal_BIOMAP_Supplementary_Tables.pdf]

Supplementary Tables

Contents

S1 Metric tables for Logistic Regression models **3**
S1.1 Binary 3
S1.2 Multiclass 4

S2 Metrics tables for Support Vector Machine models **5**
S2.1 Binary 5
S2.2 Multiclass 6

S3 Metric tables for XGBoost models **7**
S3.1 Binary 7
S3.2 Multiclass 8

S4 Sample counts for independent datasets with adenoma samples **8**

S5 Metric table for neural network model on TCGA testset **9**

S6 Metrics table for multiclass XGBoost on independent data **9**

S7 Metrics table for neural network model on independent data **9**

S8 Performance comparison to Hao 2017 **9**

S9 Performance comparison to Ibrahim 2022 **10**

S10 Performance comparison to Zheng 2020 **10**

S11 Performance comparison to Modhurkur 2021 **10**

S12 Number of normal and cancer samples from TCGA **10**

S13 Number of normal and cancer samples for the independent data sets **10**

Abbreviations	
BLCA	Bladder Urothelial Carcinoma
BRCA	Breast invasive carcinoma
COAD	Colon adenocarcinoma
ESCA	Esophageal carcinoma
HNSC	Head and Neck squamous cell carcinoma
KIRC	Kidney renal clear cell carcinoma
KIRP	Kidney renal papillary cell carcinoma
LIHC	Liver hepatocellular carcinoma
LUAD	Lung adenocarcinoma
LUSC	Lung squamous cell carcinoma
PRAD	Prostate adenocarcinoma
THCA	Thyroid carcinoma
UCEC	Uterine Corpus Endometrial Carcinoma

S1 Metric tables for Logistic Regression models

S1.1 Binary

a. BLCA

	Normal	BLCA
Precision	1.0	1.0
Recall	1.0	1.0
F_1	1.0	1.0
Accuracy	1.0	
MCC	1.0	

b. BRCA

	Normal	BRCA
Precision	0.957	0.99
Recall	0.917	0.995
F_1	0.936	0.992
Accuracy	0.987	
MCC	0.929	

c. COAD

	Normal	COAD
Precision	1.0	1.0
Recall	1.0	1.0
F_1	1.0	1.0
Accuracy	1.0	
MCC	1.0	

d. ESCA

	Normal	ESCA
Precision	1.0	1.0
Recall	1.0	1.0
F_1	1.0	1.0
Accuracy	1.0	
MCC	1.0	

e. HNSC

	Normal	HNSC
Precision	1.0	0.992
Recall	0.923	1.0
F_1	0.96	0.996
Accuracy	0.993	
MCC	0.957	

f. KIRC

	Normal	KIRC
Precision	1.0	1.0
Recall	1.0	1.0
F_1	1.0	1.0
Accuracy	1.0	
MCC	1.0	

g. KIRP

	Normal	KIRP
Precision	0.917	1.0
Recall	1.0	0.986
F_1	0.957	0.993
Accuracy	0.988	
MCC	0.951	

h. LIHC

	Normal	LIHC
Precision	0.929	1.0
Recall	1.0	0.989
F_1	0.963	0.995
Accuracy	0.991	
MCC	0.959	

i. LUAD

	Normal	LUAD
Precision	0.889	1.0
Recall	1.0	0.992
F_1	0.941	0.996
Accuracy	0.992	
MCC	0.939	

j. LUSC

	Normal	LUSC
Precision	0.917	1.0
Recall	1.0	0.989
F_1	0.957	0.995
Accuracy	0.99	
MCC	0.952	

k. PRAD

	Normal	PRAD
Precision	1.0	0.992
Recall	0.923	1.0
F_1	0.96	0.996
Accuracy	0.993	
MCC	0.957	

l. THCA

	Normal	THCA
Precision	0.737	1.0
Recall	1.0	0.961
F_1	0.848	0.98
Accuracy	0.965	
MCC	0.842	

m. UCEC

	Normal	UCEC
Precision	1.0	1.0
Recall	1.0	1.0
F_1	1.0	1.0
Accuracy	1.0	
MCC	1.0	

S1.2 Multiclass

	Normal	BLCA	BRCA	COAD	ESCA	HNSC	KIRC	KIRP	LIHC	LUAD	LUSC	PRAD	THCA	UCEC
precision	0.953	0.99	0.98	0.988	0.956	0.949	0.975	0.957	1.0	0.983	0.977	0.954	0.992	1.0
recall	0.937	0.99	0.99	1.0	0.935	0.977	0.963	0.957	0.968	1.0	0.914	0.984	1.0	1.0
f1	0.945	0.99	0.985	0.994	0.945	0.963	0.969	0.957	0.984	0.992	0.944	0.969	0.996	1.0
accuracy	0.975													
mcc	0.973													

Performance metrics for the logistic regression multiclass model

S2 Metrics tables for Support Vector Machine models

S2.1 Binary

a. BLCA

	Normal	BLCA
Precision	1.0	1.0
Recall	1.0	1.0
F_1	1.0	1.0
Accuracy	1.0	
MCC	1.0	

b. BRCA

	Normal	BRCA
Precision	0.957	0.99
Recall	0.917	0.995
F_1	0.936	0.992
Accuracy	0.987	
MCC	0.929	

c. COAD

	Normal	COAD
Precision	1.0	1.0
Recall	1.0	1.0
F_1	1.0	1.0
Accuracy	1.0	
MCC	1.0	

d. ESCA

	Normal	ESCA
Precision	1.0	1.0
Recall	1.0	1.0
F_1	1.0	1.0
Accuracy	1.0	
MCC	1.0	

e. HNSC

	Normal	HNSC
Precision	1.0	0.992
Recall	0.923	1.0
F_1	0.96	0.996
Accuracy	0.993	
MCC	0.957	

f. KIRC

	Normal	KIRC
Precision	1.0	1.0
Recall	1.0	1.0
F_1	1.0	1.0
Accuracy	1.0	
MCC	1.0	

g. KIRP

	Normal	KIRP
Precision	1.0	1.0
Recall	1.0	1.0
F_1	1.0	1.0
Accuracy	1.0	
MCC	1.0	

h. LIHC

	Normal	LIHC
Precision	0.929	1.0
Recall	1.0	0.989
F_1	0.963	0.995
Accuracy	0.991	
MCC	0.959	

i. LUAD

	Normal	LUAD
Precision	0.889	1.0
Recall	1.0	0.992
F_1	0.941	0.996
Accuracy	0.992	
MCC	0.939	

j. LUSC

	Normal	LUSC
Precision	0.917	1.0
Recall	1.0	0.989
F_1	0.957	0.995
Accuracy	0.99	
MCC	0.952	

k. PRAD

	Normal	PRAD
Precision	1.0	0.992
Recall	0.923	1.0
F_1	0.96	0.996
Accuracy	0.993	
MCC	0.957	

l. THCA

	Normal	THCA
Precision	0.778	1.0
Recall	1.0	0.969
F_1	0.875	0.984
Accuracy	0.972	
MCC	0.868	

m. UCEC

	Normal	UCEC
Precision	1.0	1.0
Recall	1.0	1.0
F_1	1.0	1.0
Accuracy	1.0	
MCC	1.0	

S2.2 Multiclass

	Normal	BLCA	BRCA	COAD	ESCA	HNSC	KIRC	KIRP	LIHC	LUAD	LUSC	PRAD	THCA	UCEC
Precision	0.915	0.953	0.975	1.0	0.941	0.899	0.975	0.971	1.0	1.0	0.925	0.946	0.977	0.991
Recall	0.926	0.981	0.99	1.0	0.696	0.947	0.963	0.957	0.937	0.992	0.925	0.968	0.992	1.0
F_1	0.92	0.967	0.982	1.0	0.8	0.923	0.969	0.964	0.967	0.996	0.925	0.957	0.985	0.995
Accuracy	0.96													
MCC	0.956													

Performance metrics for the Support Vector Machine multiclass model

S3 Metric tables for XGBoost models

S3.1 Binary

a. BLCA

	Normal	BLCA
Precision	1.0	0.981
Recall	0.6	1.0
F_1	0.75	0.99
Accuracy	0.982	
MCC	0.767	

b. BRCA

	Normal	BRCA
Precision	1.0	0.99
Recall	0.917	1.0
F_1	0.957	0.995
Accuracy	0.991	
MCC	0.953	

c. COAD

	Normal	COAD
Precision	1.0	1.0
Recall	1.0	1.0
F_1	1.0	1.0
Accuracy	1.0	
MCC	1.0	

d. ESCA

	Normal	ESCA
Precision	1.0	0.959
Recall	0.5	1.0
F_1	0.667	0.979
Accuracy	0.961	
MCC	0.693	

e. HNSC

	Normal	HNSC
Precision	0.923	1.0
Recall	1.0	0.992
F_1	0.96	0.996
Accuracy	0.993	
MCC	0.957	

f. KIRC

	Normal	KIRC
Precision	1.0	1.0
Recall	1.0	1.0
F_1	1.0	1.0
Accuracy	1.0	
MCC	1.0	

g. KIRP

	Normal	KIRP
Precision	0.833	0.986
Recall	0.909	0.971
F_1	0.87	0.978
Accuracy	0.963	
MCC	0.849	

h. LIHC

	Normal	LIHC
Precision	1.0	0.99
Recall	0.923	1.0
F_1	0.96	0.995
Accuracy	0.991	
MCC	0.956	

i. LUAD

	Normal	LUAD
Precision	1.0	1.0
Recall	1.0	1.0
F_1	1.0	1.0
Accuracy	1.0	
MCC	1.0	

j. LUSC

	Normal	LUSC
Precision	1.0	1.0
Recall	1.0	1.0
F_1	1.0	1.0
Accuracy	1.0	
MCC	1.0	

k. PRAD

	Normal	PRAD
Precision	0.75	0.992
Recall	0.923	0.968
F_1	0.828	0.98
Accuracy	0.964	
MCC	0.813	

l. THCA

	Normal	THCA
Precision	1.0	0.992
Recall	0.929	1.0
F_1	0.963	0.996
Accuracy	0.993	
MCC	0.96	

m. UCEC

	Normal	UCEC
Precision	1.0	1.0
Recall	1.0	1.0
F_1	1.0	1.0
Accuracy	1.0	
MCC	1.0	

S3.2 Multiclass

	Normal	BLCA	BRCA	COAD	ESCA	HNSC	KIRC	KIRP	LIHC	LUAD	LUSC	PRAD	THCA	UCEC
Precision	0.954	0.981	0.985	1.0	0.979	0.985	0.942	1.0	1.0	1.0	0.978	0.984	0.984	0.991
Recall	0.943	0.981	0.995	0.987	1.0	0.985	1.0	0.928	0.989	1.0	0.978	0.984	0.984	1.0
F_1	0.949	0.981	0.99	0.994	0.989	0.985	0.97	0.962	0.995	1.0	0.978	0.984	0.984	0.995
Accuracy	0.982													
MCC	0.980													

Performance metrics for the XGBoost multiclass model

S4 Sample counts for independent datasets with adenoma samples

	COAD	THCA
Normal	11	50
Adenoma	9	17
Cancer	9	74

The number of normal, adenoma and cancer samples for the independent datasets that contain adenoma samples (COAD and THCA)

S5 Metric table for neural network model on TCGA testset

	Normal	BLCA	BRCA	COAD	ESCA	HNSC	KIRC	KIRP	LIHC	LUAD	LUSC	PRAD	THCA	UCEC
Precision	0.958	0.99	0.98	0.988	0.978	0.963	0.963	0.957	1.0	1.0	1.0	0.947	0.985	1.0
Recall	0.92	0.99	0.98	1.0	0.978	0.992	0.963	0.971	0.979	1.0	0.957	0.984	1.0	1.0
F_1	0.939	0.99	0.98	0.994	0.978	0.978	0.963	0.964	0.989	1.0	0.978	0.965	0.992	1.0
Accuracy							0.978							
MCC							0.976							

Performance metrics for the neural network model on the TCGA testset

S6 Metrics table for multiclass XGBoost on independent data

	Normal	BRCA	COAD	ESCA	HNSC	KIRC	LIHC	PRAD	THCA
Precision	0.922	0.971	0.318	0.902	1.0	1.0	1.0	0.98	0.861
Recall	0.803	0.85	0.778	0.925	0.167	0.348	1.0	0.251	0.838
F_1	0.858	0.907	0.452	0.914	0.286	0.516	1.0	0.4	0.849
Accuracy					0.68				
MCC					0.661				

Performance metrics for the multiclass XGBoost model on the independent data sets

S7 Metrics table for neural network model on independent data

	Normal	BRCA	COAD	ESCA	HNSC	KIRC	LIHC	PRAD	THCA
Precision	0.957	1.0	0.368	0.955	1.0	0.971	0.985	1.0	0.857
Recall	0.845	0.825	0.778	0.96	0.119	0.717	1.0	0.955	0.892
F_1	0.897	0.904	0.5	0.958	0.213	0.825	0.992	0.977	0.874
Accuracy					0.867				
MCC					0.844				

Performance metrics for the multiclass DNN model on the independent data sets

S8 Performance comparison to Hao 2017

	Model	BRCA	COAD	LIHC	LUAD
Precision	Hao 2017	0.9817	0.9485	0.9714	0.9767
	EMethylNet	0.9798	0.9875	1.0000	1.000
Recall	Hao 2017	0.9926	1.0000	0.9855	0.9921
	EMethylNet	0.9798	1.0000	0.9789	1.000

Test set performance comparison to Hao 2017. Precision and recall calculated from their reported confusion matrix.

S9 Performance comparison to Ibrahim 2022

	Model	BLCA	BRCA	ESCA	HNSC	KIRC	KIRP	LIHC	LUAD	LUSC	PRAD	THCA	UCEC
ROC AUC	Ibrahim 2022	0.945	0.955	0.874	0.974	0.98	0.98	0.998	0.938	0.924	0.999	1	0.998
	EMethylNet	0.9998	0.9999	0.9994	0.9999	0.9998	0.9994	1.0000	1.0000	0.9996	0.9993	0.9999	1.0000

Test set performance comparison to Ibrahim 2022. Ibrahim reported scores rounded to three decimal places.

S10 Performance comparison to Zheng 2020

	Model	BLCA	BRCA	COAD	ESCA	HNSC	LIHC	PRAD	THCA
ROC AUC	Zheng 2020	1	1	1	0.98	0.99	1	1	1
	EMethylNet	0.9998	0.9999	1.000	0.9994	0.9999	1.0000	0.9993	0.9999
Precision	Zheng 2020	0.9759	0.9810	1.0000	0.6579	0.9619	0.9851	1.0000	1.0000
	EMethylNet	0.9904	0.9798	0.9875	0.9783	0.9632	1.0000	0.9466	0.9847
Recall	Zheng 2020	0.9878	1.0000	0.9861	0.6410	0.9099	0.9851	1.0000	1.0000
	EMethylNet	0.9904	0.9798	1.0000	0.9783	0.9924	0.9789	0.9841	1.0000

Test set performance comparison to Zheng 2020. Zheng 2020 reported ROC AUCs rounded to two decimal places. Precision and Recall values retrieved from the GitHub repository of Zheng 2020.

S11 Performance comparison to Modhurkur 2021

	Model	BLCA	BRCA	COAD	ESCA	HNSC	KIRC	KIRP	LIHC	LUAD	LUSC	PRAD	THCA	UCEC
Precision	Modhurkur 2021	0.994	0.976	0.993	0.982	0.921	1	0.986	1	0.993	0.91	1	1	0.987
	EMethylNet	0.9904	0.9798	0.9875	0.9783	0.9632	0.9630	0.9571	1.0000	1.0000	1.0000	0.9466	0.9847	1.0000
Recall	Modhurkur 2021	0.956	0.932	0.98	0.921	0.926	0.974	1	0.993	0.993	0.953	0.98	1	0.97
	EMethylNet	0.9904	0.9798	1.0000	0.9783	0.9924	0.9630	0.9710	0.9789	1.0000	0.9570	0.9841	1.0000	1.0000
F1	Modhurkur 2021	0.974	0.954	0.987	0.951	0.924	0.987	0.993	0.996	0.993	0.931	0.989	1	0.978
	EMethylNet	0.9904	0.9798	0.9937	0.9783	0.9776	0.9630	0.9640	0.9894	1.0000	0.9780	0.9650	0.9923	1.000

Test set performance comparison to Modhurkur 2021. Modhurkur reported scores to three decimal places.

S12 Number of normal and cancer samples from TCGA

	BLCA	BRCA	COAD	ESCA	HNSC	KIRC	KIRP	LIHC	LUAD	LUSC	PRAD	THCA	UCEC	**Total**
Normal	21	96	38	16	50	160	45	50	32	42	50	56	46	**702**
Cancer	415	793	315	186	530	325	276	380	475	370	503	515	439	**5522**
Total	436	889	353	202	580	485	321	430	507	412	553	571	485	**6224**

Number of normal and cancer samples for the TCGA dataset

S13 Number of normal and cancer samples for the independent data sets

	BRCA	COAD	ESCA	ESCA 2	HNSC	KIRC	LIHC	PRAD	THCA	**Total**
Normal	17	20	75	39	0	46	0	0	67	**264**
Cancer	40	9	125	75	42	46	66	199	74	**676**
Total	57	29	200	114	42	92	66	199	141	**940**

Number of normal and cancer samples for the independent data sets
