# Supplementary material for: Early detection and diagnosis of cancer with interpretable machine learning to uncover cancer-specific DNA methylation patterns
Source: Biol Methods Protoc. 2024 Jun 20;9(1):bpae028. doi: 10.1093/biomethods/bpae028 (PMC11186673; doi:10.1093/biomethods/bpae028)
Supplement: bpae028_Supplementary_Data [file bpae028_supplementary_data.zip › EMethylNet_Newsham_etal_BIOMAP_Supplementary_Figures.pdf]

Supplementary Figures

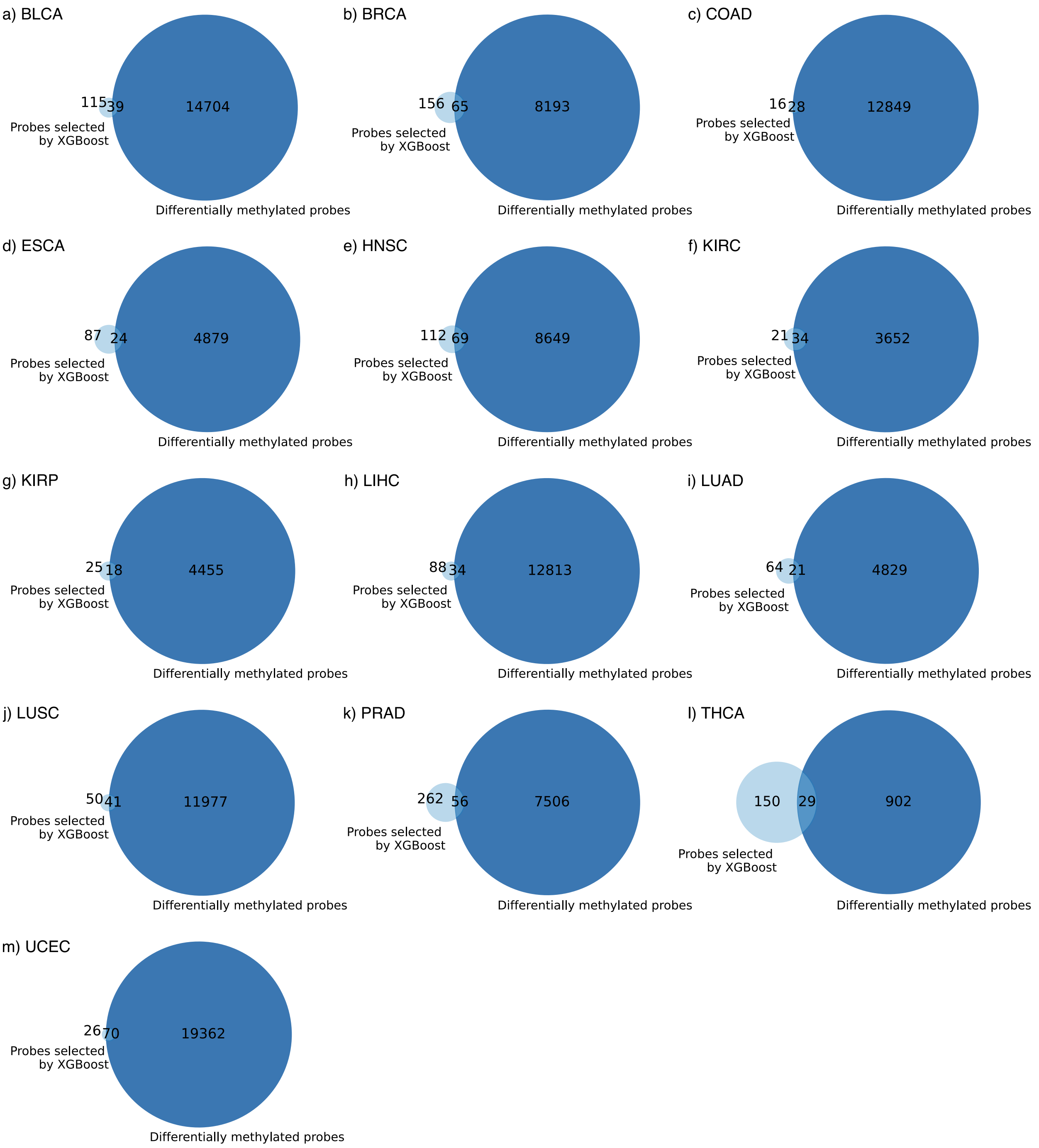

Figure S1: Venn diagrams showing the number of probes overlapping between differentially methylated probes and PCCs from the XGBoost binary models.

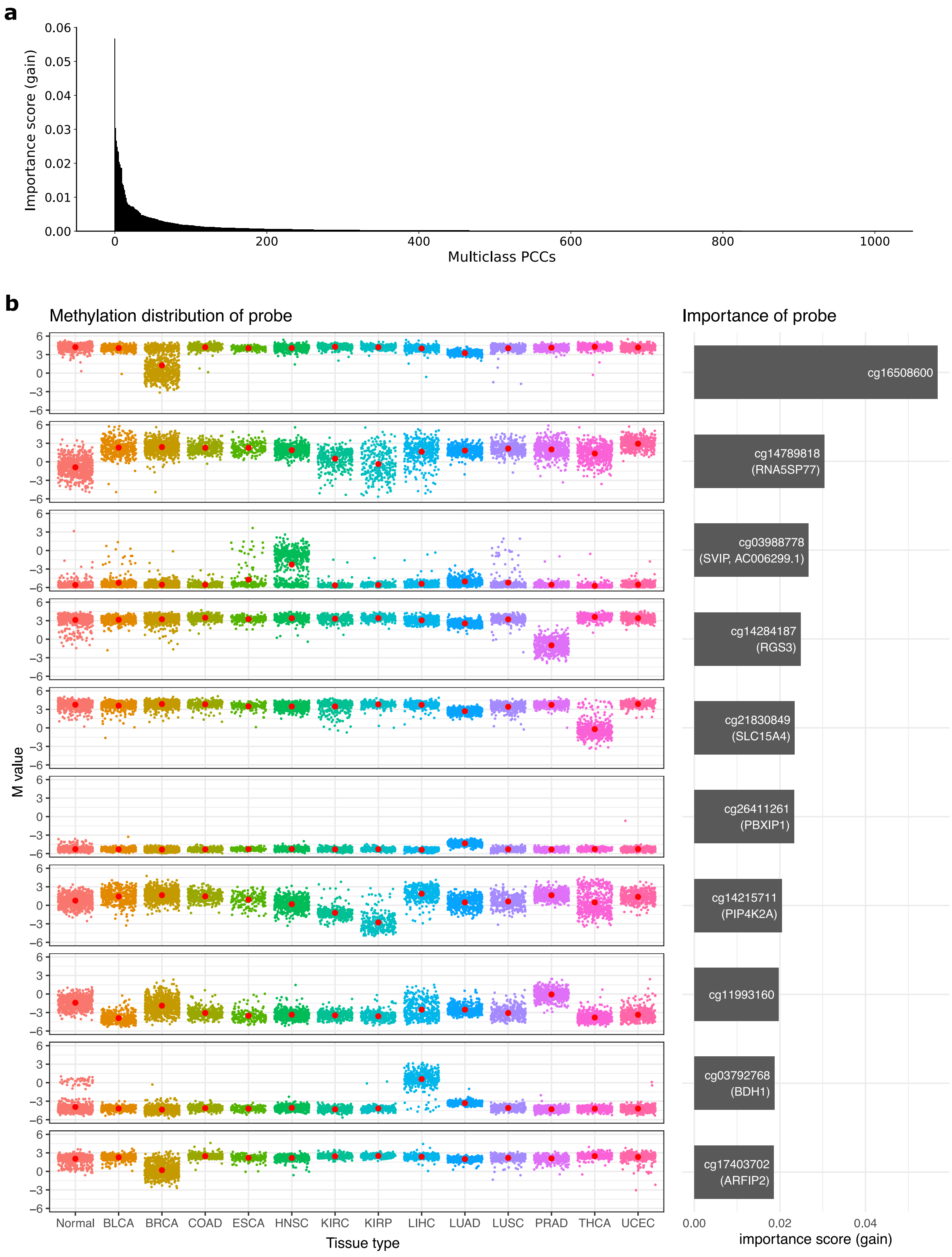

Figure S2: Feature importance of the most important PCCs. **a** The feature importance scores of the top 1000 PCCs from the multiclass XGBoost model. **b** The methylation distribution for the top 10 PCCs, for all cancer types and the normal class. The importance score, probe name and gene name(s) (if the probe maps to a gene) is visualised on the right. The red dot indicates the mean value.

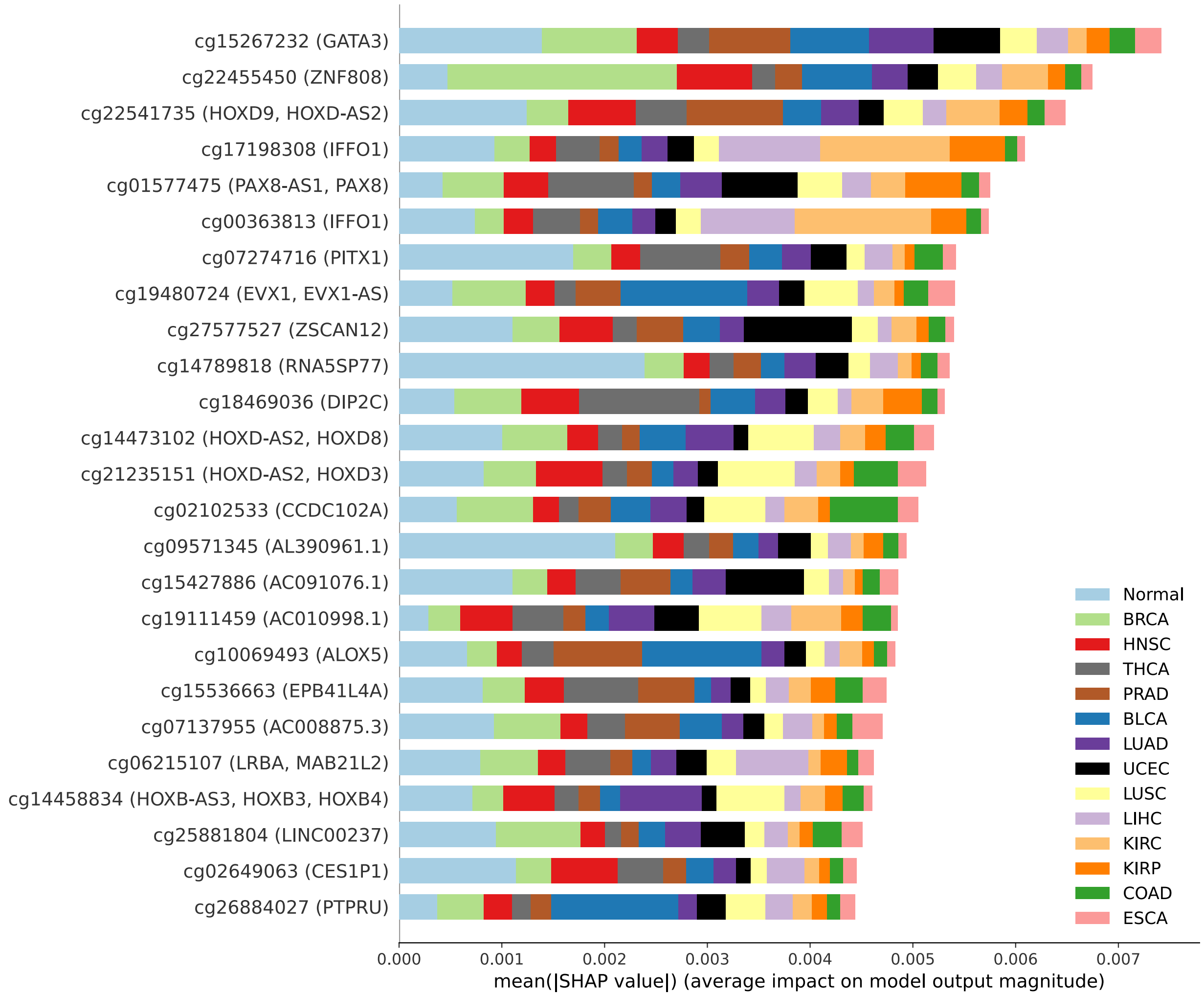

Figure S3: Average absolute SHAP values of the multiclass DNN across all classes, for the 25 features with the highest values. Colour indicates the class, and the genes that the features map to are given in brackets.

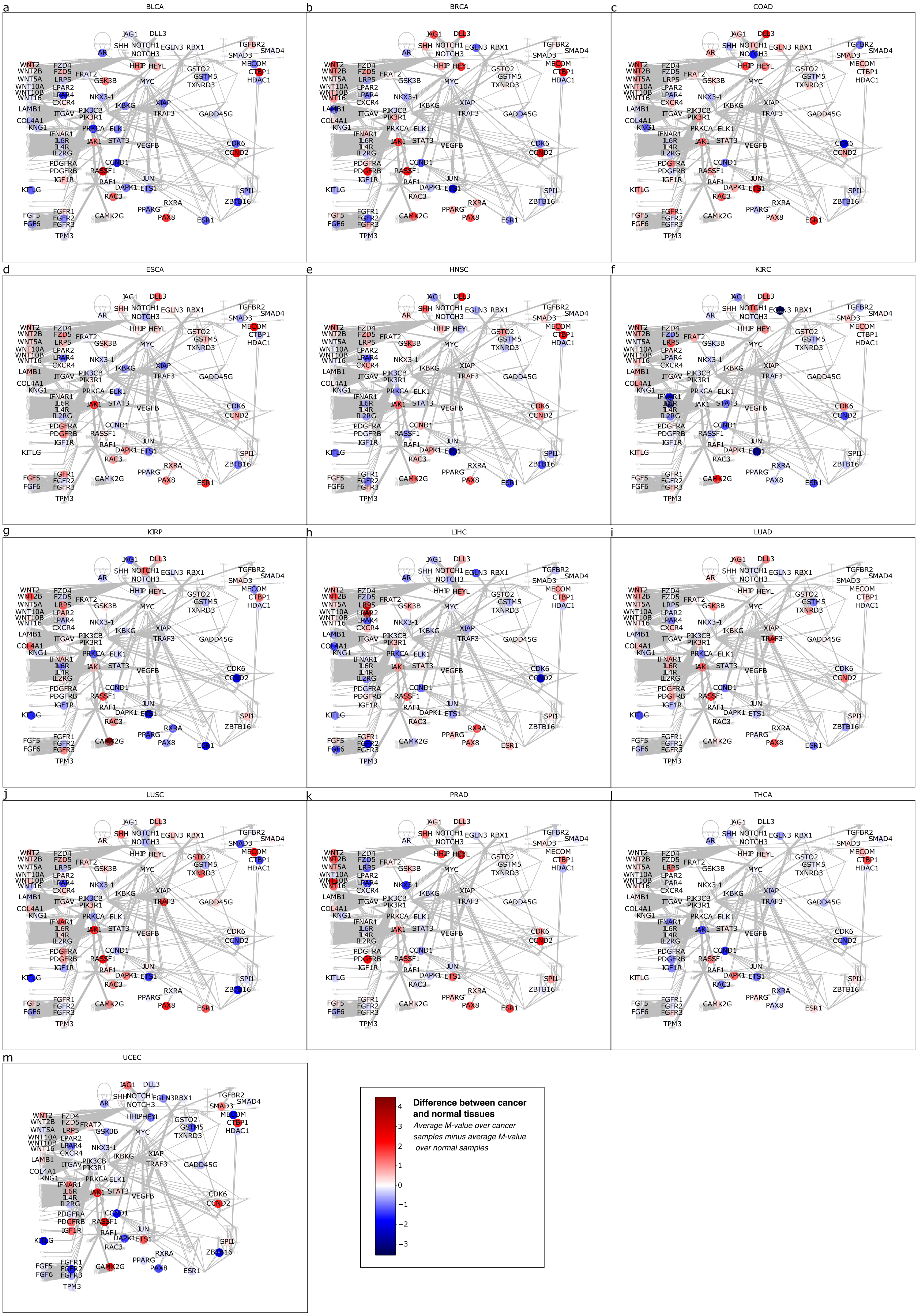

Figure S4: Visualisations of the KEGG *Pathways in cancer* pathway for all cancer types, where the colour represents the difference in methylation between cancer and normal tissues. Only the multiclass genes in the pathway are shown.

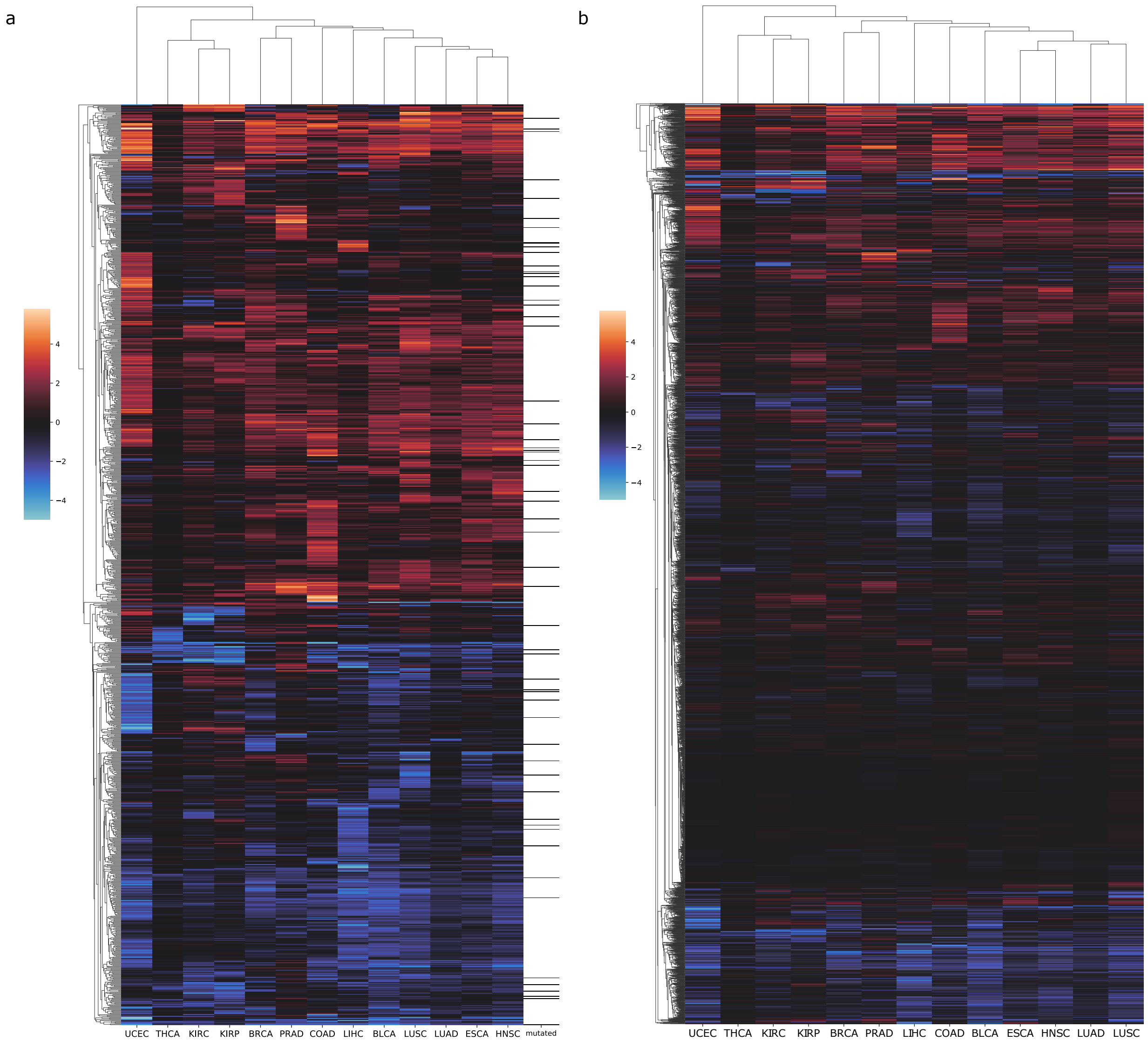

Figure S5: Heatmaps showing the difference in methylation between cancer and normal for all cancer types. **a** Only showing the PCCs with an absolute difference greater than 2. The rightmost column displays whether the feature maps to a gene that is found to be mutated in the COSMIC Cancer Gene Census or the TCGA Significantly Mutated Gene list. **b** All 3388 PCCs are shown.

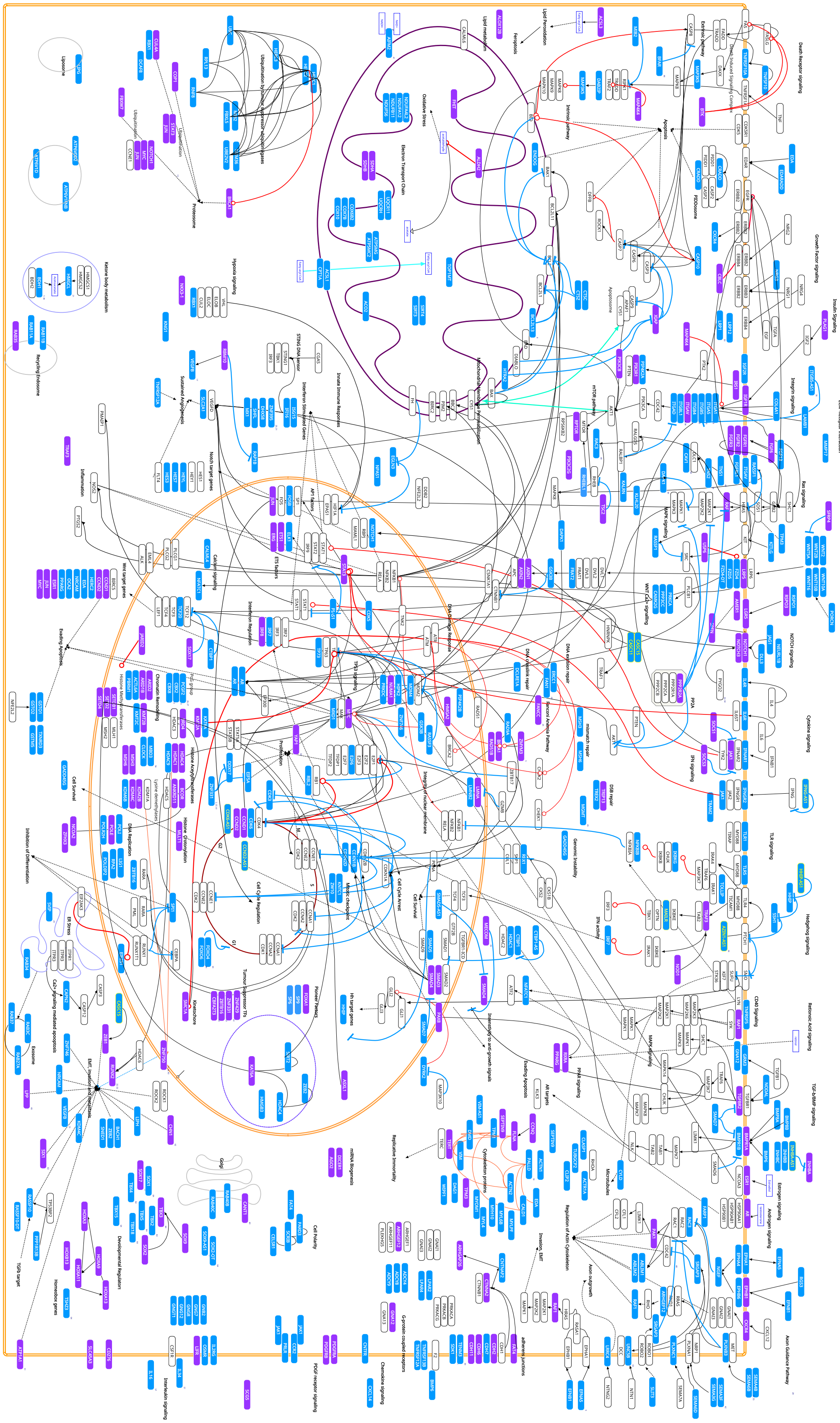

Figure S6: The Pan-Cancer Methylome Network showing cancer associated pathways and processes affected by changes in DNA methylation identified in this study. Nodes associated with multiclass PCC associated genes are coloured in blue and cancer associated (from cancer gene census) PCC genes are coloured purple. Interaction colour codes are as follows: Red lines - catalytic, blue - inhibitory, light green - translocations, and black - physical interactions, regulation or signaling events.

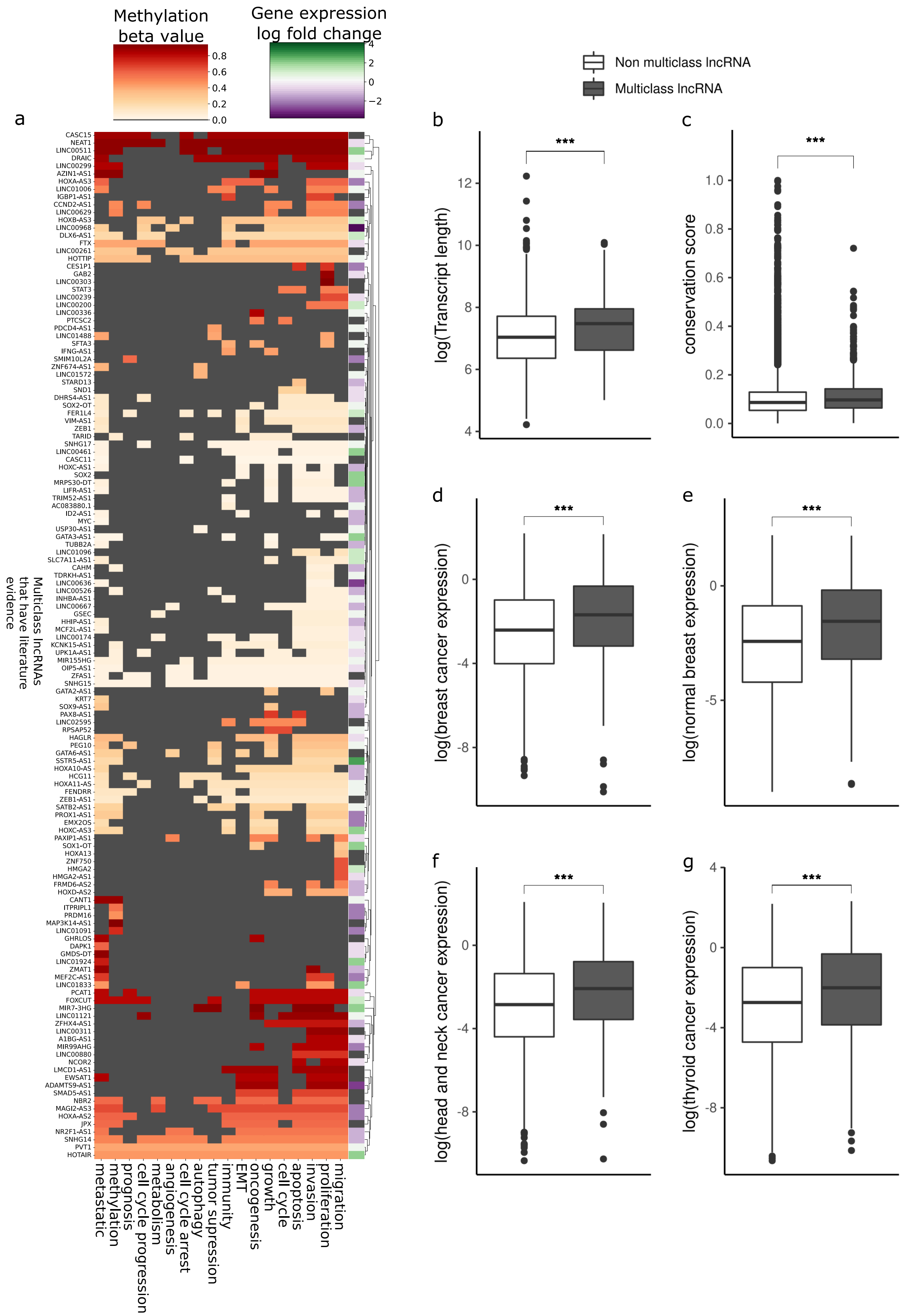

Figure S7: Literature evidence and features of the multiclass lncRNAs. **a** A heatmap of breast cancer data showing the average beta value of the multiclass lncRNAs with literature evidence, and the cancer hallmarks they are associated with. The lncRNA gene symbols are shown. The row annotation indicates the log fold change from differential expression analysis, where non-significant fold change (adjusted p-value>0.05) is in grey. **b-g** Boxplots of various features of the non multiclass lncRNAs and multiclass lncRNAs. '***' indicates p-value < 0.001. The following features are shown: $\log_e$ transcript length (**b**), conservation (**c**), $\log_e$ breast cancer expression (**d**), $\log_e$ normal breast expression (**e**), $\log_e$ head and neck cancer expression (**f**) and $\log_e$ thyroid cancer expression (**g**).

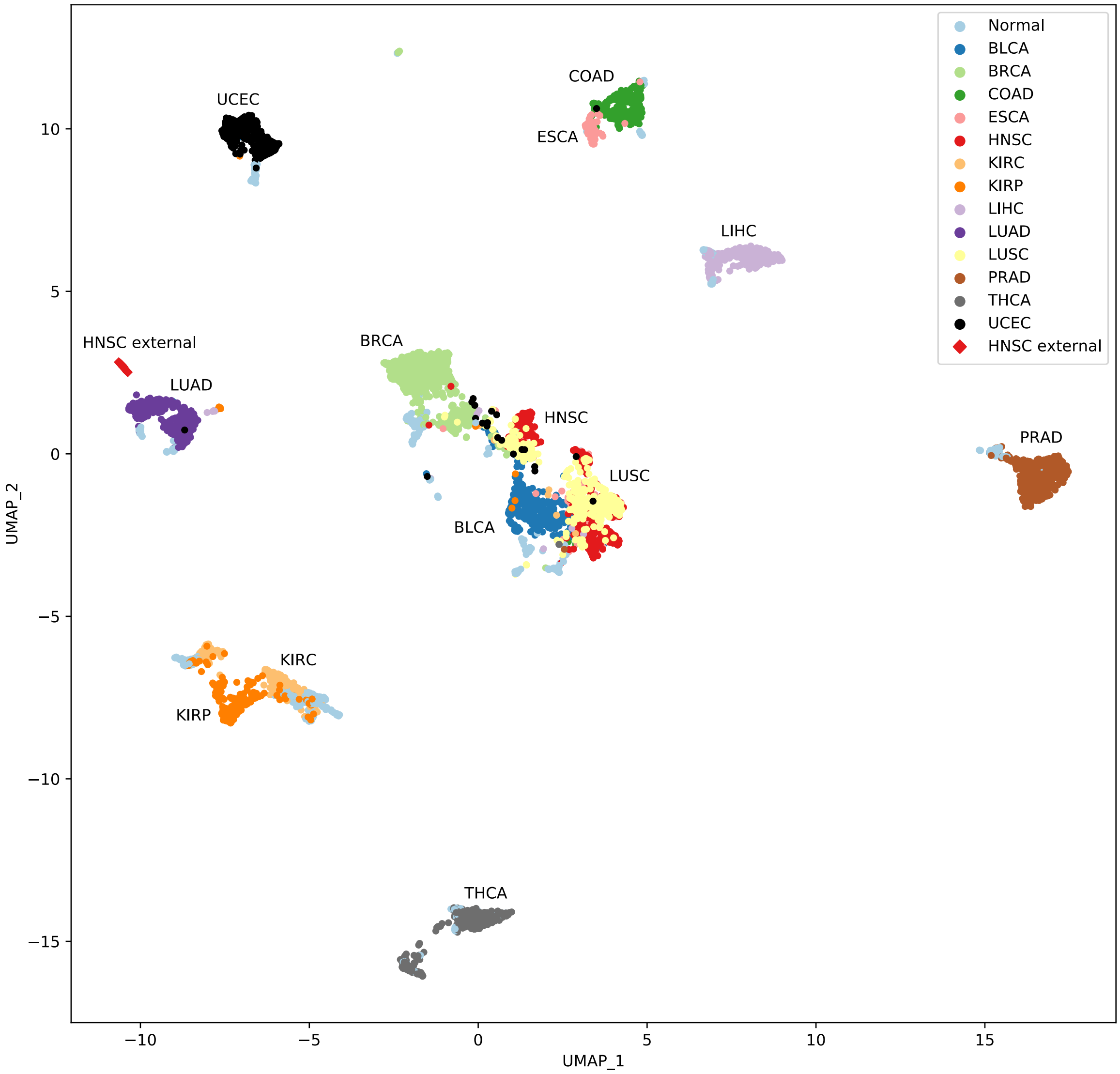

Figure S8: UMAP visualisation of all preprocessed TCGA data and the independent HNSC data. Colour represents the cancer type, and the main cluster of each cancer type is annotated.
